# Formation and suppression of acoustic memories during human sleep

**DOI:** 10.1038/s41467-017-00071-z

**Published:** 2017-08-08

**Authors:** Thomas Andrillon, Daniel Pressnitzer, Damien Léger, Sid Kouider

**Affiliations:** 1 Brain and Consciousness Group (ENS, EHESS, CNRS), Département d’Études Cognitives, École Normale Supérieure-PSL Research University, Paris, 75005 France; 2 École Doctorale Cerveau Cognition Comportement, Université Pierre et Marie Curie, Paris, 75005 France; 3 Laboratoire des Systèmes Perceptifs, CNRS UMR 8248, Département d’Études Cognitives, École Normale Supérieure-PSL Research University, Paris, 75005 France; 4 Université Paris Descartes, Sorbonne Paris Cité, APHP, Hôtel Dieu, Centre du Sommeil et de la Vigilance et EA 7330 VIFASOM, Paris, 75006 France

## Abstract

Sleep and memory are deeply related, but the nature of the neuroplastic processes induced by sleep remains unclear. Here, we report that memory traces can be both formed or suppressed during sleep, depending on sleep phase. We played samples of acoustic noise to sleeping human listeners. Repeated exposure to a novel noise during Rapid Eye Movements (REM) or light non-REM (NREM) sleep leads to improvements in behavioral performance upon awakening. Strikingly, the same exposure during deep NREM sleep leads to impaired performance upon awakening. Electroencephalographic markers of learning extracted during sleep confirm a dissociation between sleep facilitating memory formation (light NREM and REM sleep) and sleep suppressing learning (deep NREM sleep). We can trace these neural changes back to transient sleep events, such as spindles for memory facilitation and slow waves for suppression. Thus, highly selective memory processes are active during human sleep, with intertwined episodes of facilitative and suppressive plasticity.

## Abstract (web summary)

Though memory and sleep are related, it is still unclear whether new memories can be formed during sleep. Here, authors show that people could learn new sounds during REM or light non-REM sleep, but that learning was suppressed when sounds were played during deep NREM sleep.

## Introduction

The ability to learn during sleep is both the focus of many scientific studies as well as an ancient fantasy^1^. However, well-controlled studies showing the formation of new mnesic traces during sleep remain scarce^2–8^ and coexist with numerous null results^9–11^. This paucity of positive results contrasts with the vast literature linking sleep to the consolidation of pre-existing memories^12–15^.

Current models of the relationship between sleep and memory propose specific mechanisms to account for this discrepancy. According to the active consolidation hypothesis^13, 14, 16, 17^, previously learnt information is replayed during sleep, enabling the transfer and strengthening of memories for long-term cortical storage. The gating of sensory information at the thalamic level^18^ could prevent interferences from external events in order to ensure optimal conditions for the consolidation of already existing memories. Sleep spindles have often been proposed as a mechanism ensuring such offline hippocampal–cortical dialogue^13, 19^. The reversal of the information flow between cortical and hippocampal structures could also explain the difficulty to form new memories during sleep^13, 16^.

Another influential theoretical proposal, the synaptic homeostasis hypothesis^12, 20^, proposes that sleep reflects homeostatic constraints promoting downscaling rather than potentiation of synaptic connections, leading to a dampening or suppression of existing memories^12^. Because only the strongest memories are conserved, consolidation would result from a higher signal-to-noise ratio rather than an absolute increase in memory strength^21^. Another consequence of synaptic downscaling would be an increased difficulty in forming new mnesic traces. Such account can seem, at first, opposed to the active consolidation hypothesis, as it stresses the importance of synaptic downscaling rather than synaptic potentiation during sleep. However, both types of processes could occur during sleep but during distinct phases of sleep: active consolidation in light NREM and REM sleep and synaptic downscaling in deep NREM sleep (see ref. ^22^ for a review).

What these models have in common is that they all propose mechanisms explaining how memory consolidation may negatively impact the formation of new memories. However, little is known about how sleep and its associated rhythms eventually modulate the ability to learn. Past studies revealed the surprising ability of the sleeping brain to form new memories^6, 7, 23, 24^ and to process sensory information in a complex and flexible fashion^25–28^; these results advocate for a more detailed investigation of whether and how memories can be formed during sleep. In particular, the question of how sleep stages modulate environmental learning remains unanswered.

To probe the occurrence of learning across sleep, we investigated the formation of memories for novel sensory stimuli presented overnight. We used a noise-memory paradigm,^29^ in which participants have to detect repeating noise segments embedded within running white noise. Performance usually increases with exposure to the same noise exemplar, reflecting perceptual learning over time^29^. As learning is unsupervised and occurs even in the absence of attention^30, 31^, the paradigm is appropriate to probe memory processes during sleep. The noise samples are fully novel to listeners, ensuring the formation of new memories and not consolidation. Importantly, electroencephalographic (EEG) markers of noise learning during wakefulness have been recently established^30, 31^; so EEG can be used in the absence of behavioral report during sleep. We reasoned that by providing sleepers with sensory information that can be learnt passively, we could probe neural plasticity during sleep. Indeed, so far, only conditioning had been evidenced in sleeping animals^6, 32^ or humans^3, 7, 23^ while declarative forms of memory tend to produce null results^9, 33^.

Here, we show that other forms of implicit memory can be acquired during sleep. Performance upon awakening is improved, providing evidence for perceptual learning during REM sleep. EEG markers of learning computed overnight confirm sleepers’ ability to learn in both REM sleep and light NREM sleep. The link between this form of perceptual learning and more standard instances of nondeclarative memory remains to be specified, but previous data suggests that it contains both shorter-term (hundreds of millisecond) and longer-term (from tens of seconds to weeks) components^30^. Presenting stimuli during deep NREM sleep, however, can lead to the suppression of previous learning and can even have a negative impact on subsequent learning upon awakening. In this study, we link learning or the suppression of learning to sleep hallmarks such as slow waves and sleep spindles in NREM sleep or rapid eye movements in REM sleep. Thus, both the formation and suppression of new memories can take place during sleep, depending on sleep stages and rhythms.

## Results

### Experimental Design

**Fig. 1:**
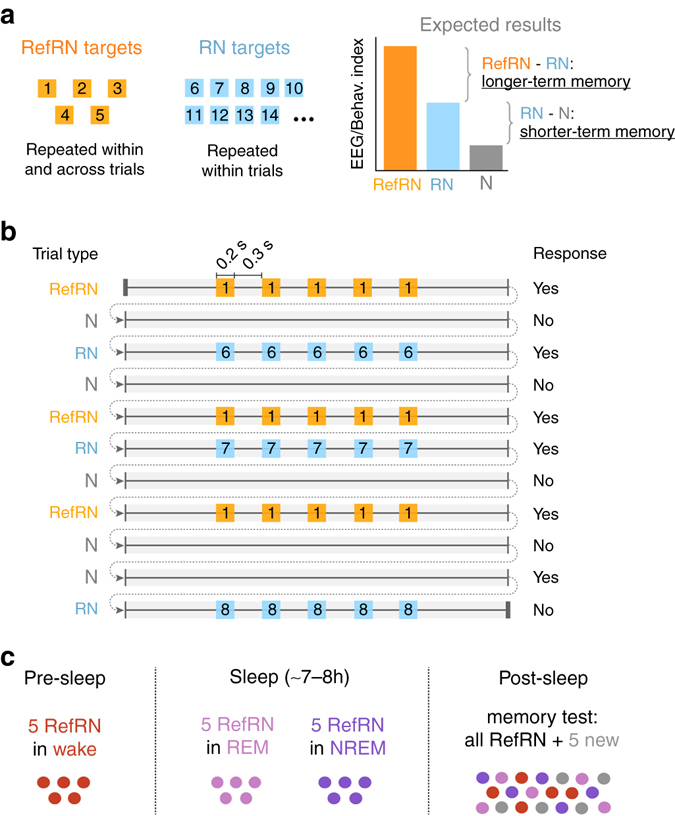
Noise memory paradigm in wakefulness and sleep. **a**, **b** Stimuli and expected results: Participants (*N* = 20) were instructed to discriminate between trials made of running white noise (N) and trials that contained a repeated pattern (RN and RefRN), made by the seamless concatenation of short (0.2 s) noise segments (targets) interleaved with 0.3 s fresh white-noise fillers. RN (within-trial repetition) and RefRN (within- and across-trial repetition) trials had an identical structure and differed only regarding the amount of exposure to the target. Participants’ ability to discriminate RN from N trials evidences shorter-term memory for the novel repeated target. A better discrimination for RefRN compared to RN trials additionally indicates longer-term memory processes (**a**, *right*). **c** Full-night recording*:*. Each recording session started with a pre-sleep phase, during which participants were instructed to remain awake. In all, 5 unique randomly generated RefRN were used in the pre-sleep phase for each participant. In the sleep phase, participants were lying on a bed while being continuously exposed to white-noise stimuli. Different sets of unique RefRN targets were used depending on participants’ vigilance states (wake, non-rapid eye movement (NREM) and REM sleep). Finally, participants were tested upon awakening (post sleep) on all RefRN targets heard during the pre-sleep and sleep phases, along with 5 novel RefRNs (memory test). Each RefRN target was played in a separate block along new RN and N trials

Human listeners were tested in a whole-night sleep experiment (Fig. 1). In a first pre-sleep phase, participants who were awake (*N* = 20) were instructed to discriminate Gaussian noise (N) from noise with embedded repeated patterns (repeated noise, RN), those patterns simply being identical snippets of Gaussian noise. Unbeknownst to the participants, five different target noise segments, which repeated within trials, also reoccurred across several trials (reference-RN, RefRN). A higher repetition detection rate for RefRN, heard across many trials, relative to RN, heard only during one trial, indicates perceptual learning^29, 30^. A subsequent sleep phase started immediately after, during which participants fell asleep while performing the task. The sound presentation continued but two new sets of five RefRN targets were introduced, respectively, during NREM and REM sleep (i.e., when participants were asleep and unresponsive). Finally, in the morning, participants started the post-sleep test with the same discrimination task as for the pre-sleep phase. Stimuli included all RefRN targets heard during the pre-sleep wake phase, NREM sleep, and REM sleep, together with a new set of five RefRN targets for baseline comparison.

**Fig. 2:**
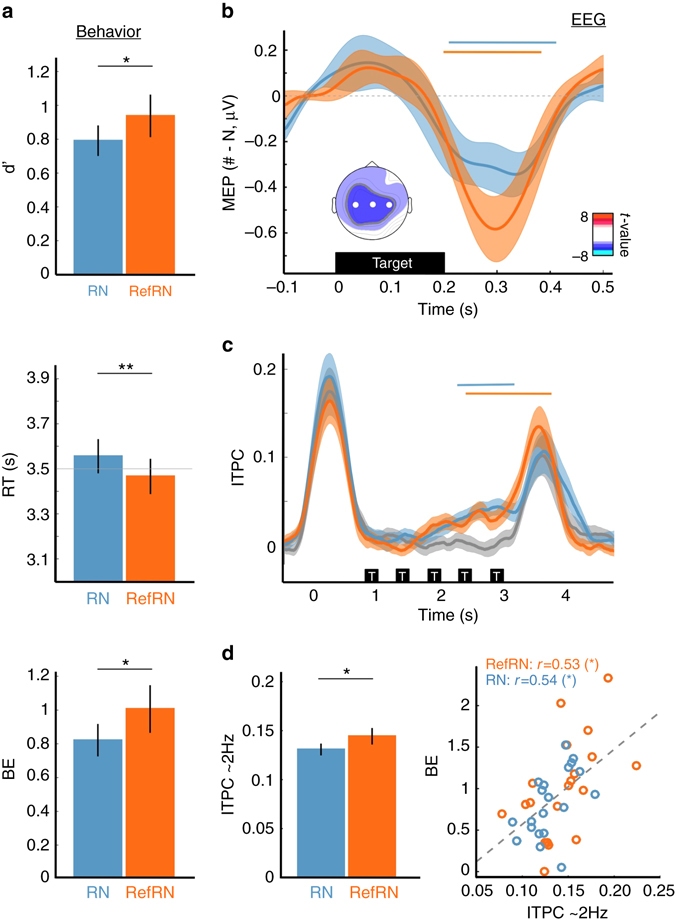
Behavioral and electrophysiological indexes of perceptual learning in wakefulness. **a** Behavioral indexes of memory for noise. Participants could discriminate RN and RefRN from noise as indicated by the positive sensitivity (*d*′, *top*). In addition, performance was better for RefRN compared to RN: *d*′ was increased while reaction times (RTs, *middle*) were decreased for RefRN. We combined these two variables into a Behavioral Efficacy (BE, *bottom*) index. Error bars denote the standard error of the mean across participants (*N* = 20). *Stars* atop graphs refer to the RefRN vs. RN comparison (paired t-test, here and below: *P* < 0.01: **; *P* < 0.05: *). **b** Target-locked memory-evoked potentials (MEPs). Averaged EEG activity time-locked to the position of targets’ onset for RefRN (*orange*), RN (*blue*) compared to N trials during the pre-sleep phase. All targets but the first one from a given trial were used to compute these MEPs (4 targets per trial). MEPs were temporally smoothed using a 50 ms-wide Gaussian kernel. Shaded areas denote the SEM across participants. *Horizontal orange* and *blue lines* show significant clusters for the RefRN vs. N (*orange*, [200, 400] ms post target) and RN vs. N (*blue*, [200, 410] ms) comparisons (*P*_cluster_ < 0.005). The inset shows the scalp topographies of *t*-values corresponding to the RefRN vs. N cluster (i.e., *t*-values obtained via a *t*-test of the RefRN vs. N difference for the MEPs waveforms averaged between 200 and 400 ms across participants). *White dots* show the central electrodes used in **b**–**d**. **c** Stimulus-locked Inter-Trial Phase Coherence (ITPC). An increase in ITPC ([1.5, 3.5] Hz) was observed for RefRN ([2.3, 3.8]s post stimulus onset, *P*_cluster_ < 0.05) and RN trials ([2.2, 3.1]s, *P*_cluster_ < 0.05) compared to N. ITPC was here corrected for baseline activity ([−1.3, −0.3]s). **d** Averaging ITPC over the stimulus presentation window ([0.8, 3.8]s) revealed higher ITPC values for RefRN values compared to RN (two-tailed paired *t*-test). ITPC was correlated with BE (*right*, Pearson’s correlation)

We replicated previous findings during the pre-sleep wake phase^29–31^. Namely, participants reliably reported targets for both the RefRN and RN conditions (Fig. 2a and Supplementary Fig. 5a). In addition, RefRNs were detected with higher sensitivity (*d*′) and shorter reaction times (RTs) compared to RNs (Fig. 2a). We combined these two behavioral measures into a Behavioral Efficacy index (BE, see Methods and ref. ^30^). An index of perceptual learning was computed by contrasting the RefRN vs. RN conditions (BE_RefRN_—BE_RN_). A greater BE was observed for RefRN targets compared to RN (paired *t*-test, *t*(19) = 2.73, *P* = 0.013, Hedges’ *g* = 0.36), showing robust perceptual learning when awake after only 16 exposures to a given RefRN.

### Learning during sleep impacts performance upon awakening

The same analysis was performed for the post-sleep test. We computed the index of perceptual learning separately for the four different sets of RefRN (i.e., pre-sleep, NREM and REM sets, plus the new post-sleep list). Because listeners can rapidly learn new RefRNs, isolating a pure effect of learning due to prior exposure should be established by focusing on initial trials (i.e., before additional learning occurs). We thus compared BEs computed over the initial 3 exposures.

For the pre-sleep list, participants exhibited significantly higher initial performance for RefRN targets compared to the new RN targets (two-tailed paired t-test: *t*(19) = 3.09, *P* = 0.0060, Hedges’ *g* = 0.58), confirming that previous exposure during wakefulness leads to a persistent form of perceptual learning^29^. We then turned to the analysis of stimuli heard during sleep, and we found a similar increase in sensitivity for REM sleep (two-tailed paired *t*-test: *t*(19) = 3.07, *P* = 0.0063, Hedges’ *g* = 0.54). This result demonstrates that perceptual learning takes place during REM sleep and transfers to wakefulness. When inspecting RefRN targets heard during NREM sleep, however, we found no trace of such an advantage (two-tailed paired t-test: *t*(19) = 0.57, *P* = 0.57, Hedges’ *g* = 0.11). We computed a Bayes factor^34^ (see Methods) to determine whether this nonsignificant effect can be interpreted as a genuine null result rather than a lack of sensitivity in our data. Consistent with the former interpretation, we observed a Bayes Factor of 8.1, indicating positive evidence for a null effect. Decomposing BE into accuracy (*d*′) and speed (RTs) confirmed this pattern of results with an increase in repetition-detection accuracy for the wake and REM lists (Supplementary Fig. 4a, b). Finally, examining BE for RefRN and RN separately (Supplementary Fig. 6) revealed that the modulations of the RefRN–RN difference across lists are due to modulations of performance for RefRN trials, whereas performance on RN trials remained rather constant. Accordingly, RN trials were equally new for all four lists while prior exposure varied for RefRN trials.

**Fig. 3:**
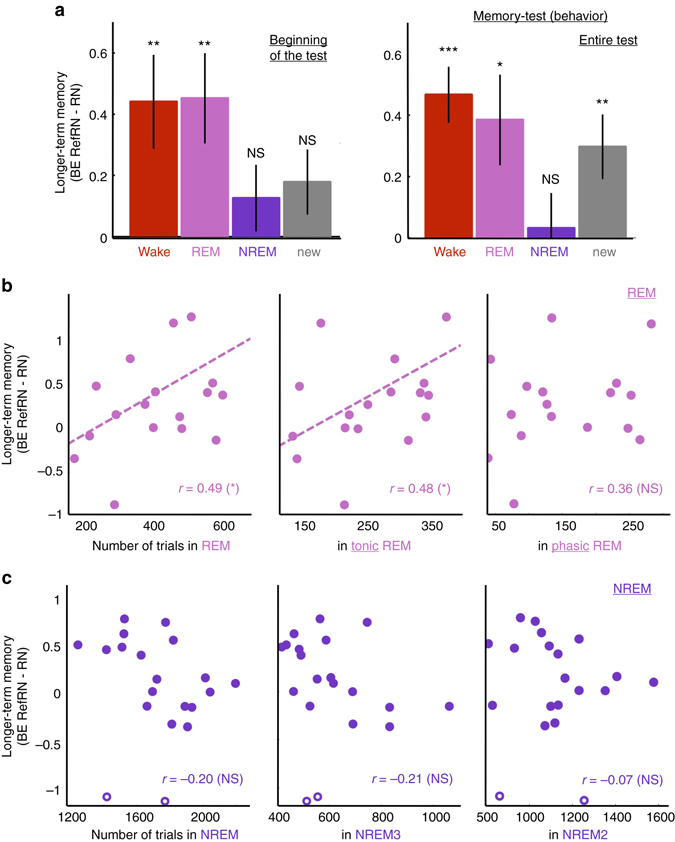
Impact of prior exposure on behavioral performance upon awakening. **a** Behavioral efficacy indexes of longer-term memory (RefRN—RN) computed for the beginning (*left*, 3 first trials) or the entire (*right*) post-sleep blocks. BE was computed separately for the RefRN heard during wakefulness, REM, NREM, or for the novel RefRN introduced in the post-sleep phase. Error bars denote the standard error of the mean across participants (*N* = 20). *Stars atop bars* indicate the results of the statistical tests (*t*-tests against 0, *P* < 0.001: ***; *P* < 0.01: **; *P* < 0.05: *, NS: *P* ≥ 0.05). Performance is better for RefRN sounds heard during wake and REM sleep at the beginning of post-sleep blocks. For the whole test analysis, all conditions improve as participants could learn even new RefRNs during the block, with the notable exception of RefRN sounds heard during NREM sleep: those were not learnt even after the whole test. **b** Correlation between the REM sleep longer-term memory index (BE_RefRN_—BE_RN_) and the number of trials played in REM sleep (*left*), tonic REM sleep (*middle*), and phasic REM sleep (*right*) across participants. **c** Correlation between the NREM sleep longer-term memory index and the number of trials played in NREM sleep (NREM2 + NREM3, *left*), NREM3 (*middle*) and NREM2 (*right*) across participants. For **b** and **c**, Pearson’s correlation coefficients are displayed on each correlation plot (*P* < 0.05: *, NS: *P* ≥ 0.05). *Open circles* (**c**) show data points detected as outliers (see Methods). The correlation coefficients obtained when excluding these data points are presented in the Results section. *Dotted lines* show the linear fit for pairs of variables with significant correlation

When examining the results over the course of the whole post-sleep test (i.e., for the 8 exposures), we found that participants remained unable to learn RefRN targets that were previously heard during NREM sleep (Bayes Factor of 34.07 suggesting strong evidence for a null effect). Strikingly, of all the noises we tested (i.e., including the novel RefRN), only the ones heard during NREM did not show any evidence of perceptual learning (Fig. 3a and Supplementary Fig. 5b). In addition, all differences between NREM effect and effects in other lists were significant (two-tailed paired t-tests, all *P* < 0.05, *N* = 20). The increase in performance for the new list can easily be interpreted as the fact that, by the end of the post-sleep phase, new RefRNs were not new anymore and had been learnt. Such learning of new RefRNs sharply contrasts with the absence of learning for NREM items. Furthermore, this pattern of results was confirmed when examining detection accuracy alone (*d*′, Supplementary Fig. 4a, b). The same pattern of results was also obtained when discarding all NREM and REM RefRN targets presented around the slightest signs of awakening (Methods and Supplementary Fig. 4c). Therefore, hearing a noise during NREM sleep made this exact same noise harder to learn upon subsequent awakening, even when compared to completely novel noises. This result reveals a suppressive effect of NREM sleep on the formation of new memory traces.

### Neurophysiological markers of wake and sleep learning

To track down the neuroplastic processes underlying perceptual learning and suppression, we analyzed EEG responses collected while participants performed the behavioral task and also while they were asleep. During the pre-sleep phase, we observed neurophysiological markers of perceptual learning consistent with behavioral findings and previous studies^30, 31^. Event-related potentials (ERPs), resembling standard auditory ERPs (Supplementary Fig. 7a and ref. ^35^), were time-locked to the repeated noise snippets (Fig. 2b, *P*_cluster_ < 0.005). Importantly, these ERPs were not locked to any salient acoustic landmark in the sounds, such as amplitude onsets or obvious spectrotemporal features (Supplementary Fig. 2). In particular, our stimuli were all constant-amplitude white noise, so no fluctuation of sound energy at the transition with the target could explain the emergence of evoked potentials. Rather, the ERPs were only present for snippets of noise that had been heard previously. Because such ERPs depend on past exposure, we termed them Memory-Evoked Potentials (MEPs^30^). These MEPs have been modeled as standard auditory potentials (N1–P2 complex) locked to idiosyncratic features within the noise, which only became salient after learning^30^. Another marker of perceptual learning is the increase in intertrial phase coherence (ITPC, Fig. 2c), which may reflect MEPs or, in addition, modulations of ongoing neural oscillations^30, 31^. We observed an increase in ITPC for RefRN targets compared to RN targets (Fig. 2d, two-tailed paired *t*-test: *t*(19) = 2.18, *P* = 0.042, Hedges’ *g* = 0.46). The ITPC increase was observed around the target presentation rate (2 Hz, Supplementary Fig. 3a) and was maximal at central electrodes (Supplementary Fig. 3b).

**Fig. 4:**
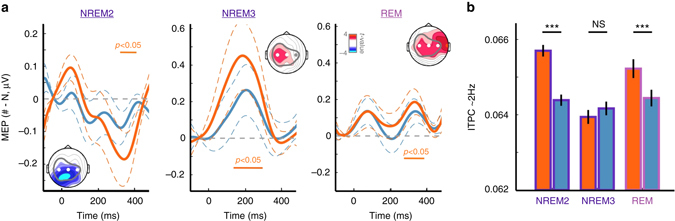
Evoked activity to repeated noise snippets during sleep. **a** Target-locked memory-evoked potentials (MEPs) in NREM2 (*left*), NREM3 (*middle*), and REM (*right*) stages of sleep for RefRN (*orange*) or RN (*blue*) trials compared to N trials. Central electrodes were used (*circles* on scalp topographies) and all targets but the first one from a given trial were used (9 targets per trial). Note the resemblance between the NREM2 MEPs and the MEPs observed in wakefulness (Fig. 2b). *Horizontal bars* show significant clusters (NREM2: ([305, 405] ms; NREM3: ([130, 300] ms; REM: [280, 390] ms; *P*_cluster_ < 0.05) for the RefRN vs. N difference (*orange*; no RN vs. N difference). *Dotted lines* denote the standard error of the mean across participants (*N* = 20). *Insets*: scalp distribution of *t*-values (RefRN vs. N, paired *t*-tests) over temporal windows corresponding to the abovementioned clusters. The *gray contour* shows the scalp distribution of the MEPs observed in wakefulness (Fig. 2b). MEPs were temporally smoothed using a 50 ms-wide Gaussian kernel. **b** Inter-trial phase coherency (ITPC) extracted over the entire night recordings (*N* = 20) on windows of 20 consecutive RefRN (*orange bars*) or RN (*blue bars*) trials. The corresponding windows were aggregated across participants (NREM2: *N* = 3698 and 3683; NREM3: *N* = 2480 and 2478; REM: *N* = 1190 and 1218 for RefRN and RN trials, respectively). ITPC was extracted around 2 Hz ([1.5, 3.5] Hz) and during stimulus presentation ([0.8, 5.5]s). Mixed-effects models revealed a significant interaction between sleep stages and stimulus condition (see Methods for details). Stars atop boxes indicate the results of *post hoc* statistical tests (*t*-tests against 0, *P* < 0.001: ***; *P* < 0.01: **; *P* < 0.05: *, NS: *P* ≥ 0.05). Note the significant increase in ITPC for RefRN compared to RN trials in stages NREM2 and REM but not in NREM3

The same MEP analyses were conducted on data collected during sleep; data from each distinct sleep phase was analyzed separately (Fig. 4a). During REM sleep, MEPs for RefRN trials differed from N trials (*P*_cluster_ < 0.05). However, the waveform of the potentials evoked by each target was different from typical MEPs (Fig. 2b). Rather, this waveform was consistent with the well-documented transformations of auditory-evoked potentials during REM sleep, which includes a decrease of N1 amplitude in favor of larger P1 and P2 potentials^36^ (Supplementary Fig. 7a). During light NREM2 sleep, RefRN trials also differed from N trials (*P*_cluster_ < 0.05). The MEPs observed for NREM2 resembled wake MEPs, with a central negative deflection. Finally, during NREM3, a difference between RefRN and N was also observed (*P*_cluster_ < 0.05) but with a much larger inter-subject variability. In addition, the waveform no longer resembled typical MEPs: the negative deflection, interpreted as an auditory N1 for wake MEPs^30^, was replaced by a positive deflection with a central scalp distribution (Fig. 4a). Again, such changes mirror the documented transformations of auditory potentials during NREM sleep^35^ (Supplementary Fig. 7a).

We completed the analyses of MEPs by examining the ITPC associated with stimulus presentation (Fig. 4b). Here, ITPC was computed on fixed-size windows of 20 consecutive RefRN or RN trials slid across the entire night recordings (see Methods). Mixed-effects modeling revealed a significant interaction between sleep stages (NREM2, NREM3, or REM) and stimulus conditions (RefRN or RN): *χ* ^2^(13) = 681.9, *P* < 2.2 × 10^−16^. *Post hoc* analyses confirmed the presence of perceptual learning during sleep. Indeed, RefRN trials elicited significantly higher ITPC levels than RN trials in NREM2 (unpaired t-test: *t*(7379) = 7.13, *P* = 1.1 × 10^−12^, Hedges’ *g* = 0.17 for 3698 and 3683 RefRN and RN windows, respectively) and REM sleep (*t*(2406) = 2.59, *P* = 0.0098, Hedges’ *g* = 0.11 for 1190 and 1218 RefRN and RN windows, respectively). However, there was no increase in ITPC for RefRN compared to RN trials in NREM3 (*t*(4956) = −0.99, *P* = 0.32, Hedges’ *g* = 0.03 for 2480 and 2478 RefRN and RN windows, respectively).

In summary, the EEG analyses during wakefulness showed clear markers of perceptual learning, replicating previous studies and consistent with behavioral findings^30, 31^. Importantly, the same EEG analyses could also be performed during sleep. There, we observed that learning was modulated by sleep stages.

### Sleep rhythms

**Fig. 5:**
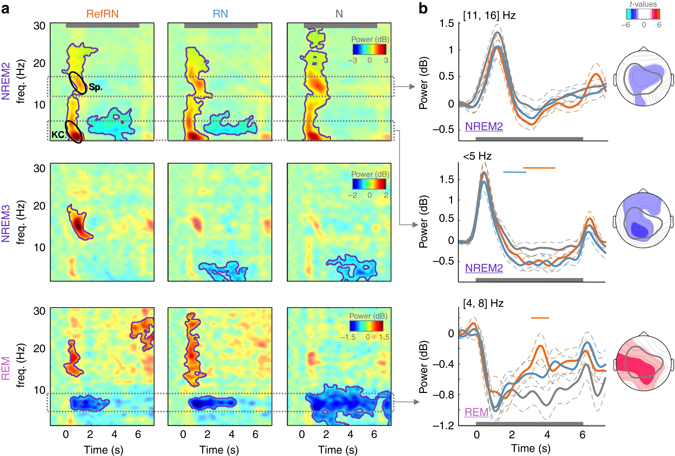
Stimulus-dependent modulations of sleep rhythms. **a** Time–frequency decomposition of the EEG signal recorded on Cz in response to RefRN (*left*), RN (*middle*), and N (*right*) trials in NREM2 (*top*), NREM3 (*middle*), and REM (*bottom*) sleep stages. Power is time-locked to stimulus onsets, averaged across participants (*N* = 20) and expressed in dB compared to a pre-stimulus baseline ([−0.25, 0]s, see Methods). Magenta contours correspond to significant modulations compared to baseline activity (cluster permutation, *P*_cluster_ < 0.05). *Gray horizontal bar* shows the stimulus presentation window. **b** Average activity in time–frequency bands typically associated to NREM (*δ* -power, < 5 Hz (corresponding to evoked KC: K-complexes,); *σ*-power, [11, 16] Hz (corresponding to Sp.: sleep spindles)) and REM rhythms (*θ*: [4, 8] Hz). The power responses were averaged over these frequency bands for NREM2 (*top*: σ, *middle*: *δ*) and REM sleep (*bottom*: *θ*). Between-condition differences are illustrated with *colored horizontal bars* (cluster-permutation test, *P*_cluster_ < 0.05, *orange*: RefRN vs. N, blue: RN vs. N). *Gray horizontal bar* shows the stimulus presentation window and *dotted lines* denote the standard error of the mean computed across participants (*N* = 20). When comparing RefRN and N trials, NREM2 was characterized by a decrease in *δ* power, REM by an increase in *θ* power for RefRN trials. Note the tendency for a decrease in power in the *σ* band for RefRN compared to N trials, although such a decrease did not resist the cluster permutation (no cluster with *P*_cluster_ < 0.05). The scalp distribution of the t-values of the RefRN vs. N comparison when averaging the power in the corresponding frequency band and over a [0.8, 5.5] s window is displayed on the side. The *gray contour* shows the scalp distribution of the MEPs observed in wakefulness (Fig. 2b). Note the overlap between the scalp distributions of the effects observed in sleep

In addition to sound-related analyses, the EEG signals were used to characterize sleep-related activity patterns so as to evaluate the impact of stimulation on sleep. We computed a time–frequency decomposition of the EEG signals, for each different sleep phases and stimulus conditions (Fig. 5). We first examined the effect of sound onset, which by construction is equivalent across all stimulus conditions (Fig. 1b and Supplementary Fig. 2). In REM sleep, sound onset robustly modulated the EEG signal within the *θ* band ([4, 8] Hz), which is a characteristic of this stage of sleep^37^, Supplementary Fig. 7b, d). In NREM2 and NREM3 stages, sound onsets were followed by evoked K complexes and sleep spindles, the hallmarks of NREM sleep^38^.

Then, we contrasted the different stimulus conditions for each sleep phase. During REM sleep, power over the *θ* band decreased over the sound presentation, more so for N trials compared to RefRN trials (*P*_cluster_ < 0.01). During NREM2 sleep, we also observed a stimulus-specific modulation of characteristic sleep rhythms (Fig. 5). RefRN trials produced a decrease in power within the slow-wave band (<5 Hz). Sleep spindles showed only a marginal modulation ([11, 16] Hz). During NREM3 sleep, no difference between conditions could be demonstrated.

Thus, in addition to EEG markers of learning, we also discovered stimulus-specific modulations of sleep rhythms in light NREM (NREM2) sleep. Interestingly, the modulations within the slow-wave and spindle bands were centrally distributed (Fig. 5b) overlapping with the topography of learning effects in wake (Fig. 2b) and did not overlap with the typical distribution of slow-wave and sleep-spindle power (Supplementary Fig. 7c). The most parsimonious interpretation is that such light NREM effects reflect local task-dependent modulations of sleep depth, accompanying the processing and learning of acoustic information. Accordingly, we observed a positive correlation (Pearson’s correlation: *r*(18) = 0.54, *P* = 0.022) between performance gain for the NREM list and sleep disruption (computed as the proportions of trials occurring during wake or NREM1 episodes), which again suggests that a decrease in sleep depth favors learning.

The behavioral and EEG analyses we have presented so far point toward a drastic difference between REM, NREM2, and NREM3 phases of sleep. However, these broadly defined phases contain a number of distinct events: REM sleep is composed of a tonic and a phasic subphase, defined by the respective absence or presence of rapid eye movements; tonic (tREM) and phasic (pREM) REM sleep have been shown to differentially impact sensory processing^39, 40^; NREM sleep contains sleep spindles and slow waves^41^, which are assumed to gate sensory processing^42^ and organize memory consolidation^12, 13^. To better understand the neural events that led to plasticity during sleep, we correlated behavioral and EEG data with these various sleep markers.

### Learning in REM sleep is predominant in the tonic phase

**Fig. 6:**
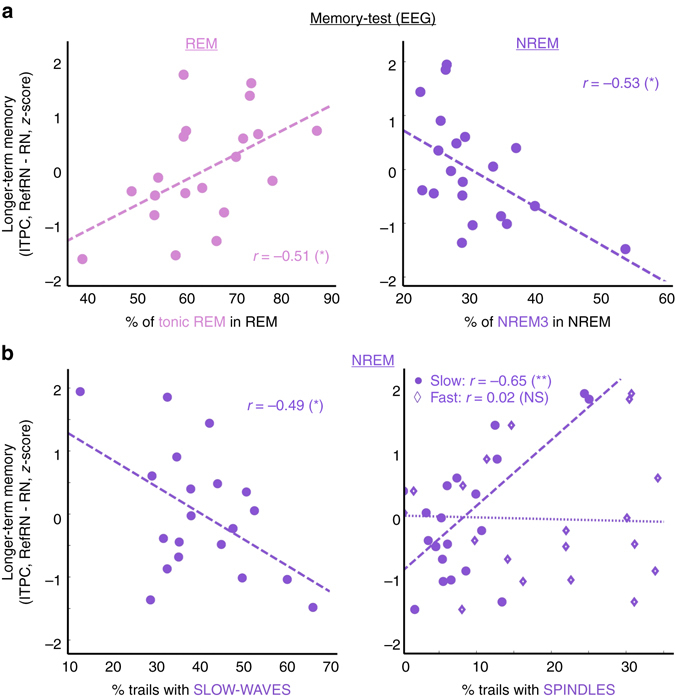
Impact of prior exposure and sleep rhythms on phase coherence upon awakening. EEG index (Inter-Trial Phase Coherence, ITPC) quantifying longer-term memory (ITPC_RefRN_—ITPC_RN_) for RefRN sounds heard during REM and NREM sleep, computed over the whole post-sleep blocks. **a** *Left:* Correlation between the magnitude of the REM sleep EEG index (*z-*scored across participants) and the proportion of trials in tonic REM sleep. *Right:* Correlation between the magnitude of the NREM sleep EEG index and the proportion of NREM3 trials within NREM sleep. As for the behavioral index (Fig. 3b), there was a positive correlation between REM sleep learning and the prevalence of tonic REM sleep. There was also a negative correlation between the marker of learning for the NREM list and the prevalence of NREM3 sleep. **b** Correlation between the magnitude of the EEG index (*z*-scored across participants) for the RefRN heard in NREM and the proportion of trials containing slow waves (*left*) or sleep spindles (*right*; circles: slow spindles; *diamonds*: fast spindles). Note the negative correlation between the EEG learning index and the proportion of trials associated to slow waves on one hand and the positive correlation with the proportion of trials associated with slow spindles on the other hand. Pearson’s correlation coefficients are displayed on each correlation plot (*P* < 0.01: **P < 0.05: *P ≥ 0.05, NS) and *dotted lines* show the linear fit between the pairs of variables

For REM sleep, we observed a positive correlation between behavioral learning (initial performance increase in the post-sleep test) and the number of trials played in REM sleep (Pearson’s correlation: *r*(18) = 0.49, *P* = 0.027). However, a stepwise regression analysis comparing the respective influence of tonic and phasic REM sleep revealed that only the number of tREM trials was predictive of the performance upon awakening (tREM: *β*(18) = 0.48, *P* = 0.034; pREM: β(18) = 0.18, *P* = 0.45; see also Fig. 3b). We replicated this pattern of results when considering neurophysiological markers of learning (Fig. 6a). The EEG index of learning (ITPC difference between RefRN and RN) was positively correlated with the proportion of tREM within REM sleep (*r*(18) = 0.51, *P* = 0.020).

These results suggest an interesting difference between tREM and pREM: within REM sleep, only tREM episodes seem conducive to stimulus-driven neuroplastic changes. However, more research is needed to confirm the respective influence of tREM and pREM on memory formation.

### Slow spindles correlate with learning in light NREM sleep

A similar correlational analysis was undertaken for NREM sleep. We investigated the role of sleep spindles, as they are thought to trigger neuronal plasticity^13^. Here, we discovered a strong and positive correlation between the percentage of trials containing slow frontal spindles and the neurophysiological markers of learning upon awakening (ITPC RefRN–RN; Pearson’s correlation coefficient: *r*(18) = 0.65, *P* = 0.0019, Fig. 6b, right). Importantly, there was no correlation when considering fast centro-parietal spindles (Pearson’s correlation coefficient: *r*(18) = 0.02, *P* = 0.92). In addition, although slow spindles are maximal at frontal electrodes, the correlation between learning and spindles’ incidence was maximal at central electrodes, overlapping again with the effects of learning observed in wakefulness and NREM2 (Supplementary Fig. 8).

### Slow waves and suppression of learning in deep NREM sleep

We attempted to trace back the suppressive effect of exposure during NREM sleep, observed on the behavioral data in the post-sleep test, to specific sleep events. Unlike in the analyses of REM sleep (Fig. 3b), there was no significant correlation between performance and the number of trials played in NREM sleep (Pearson *r*(18) = −0.20, *P* = 0.41; Fig. 3c). However, two data points (unfilled circles in Fig. 3c) were identified as outliers using the Median Absolute Deviation method^43^ (see Methods). When excluding these data points, we found a significant negative correlation between performance and the number of trials played in NREM sleep (Pearson *r*(16) = −0.55, *P* = 0.018). A stepwise regression analysis comparing the influence of NREM stages (NREM1, 2 and 3) showed that only the number of trials played in deep sleep (NREM3) was predictive performance upon awakening (β(16) = −0.50, *P* = 0.027). Nevertheless, while these results suggest that deep NREM sleep is predominantly involved in the suppressive effect of NREM exposure, it is important to stress that they were obtained only after discarding the data from two outlier participants.

The suppressive effect of deep NREM was confirmed by examining EEG responses to white-noise stimuli during the post-sleep phase (all 20 participants included, Fig. 6). The ITPC marker of learning was indeed negatively correlated with the proportion of NREM3 trials in NREM sleep (*r*(18) = −0.53, *P* = 0.016; Fig. 6a). Furthermore, as shown for other sleep rhythms, there was a negative correlation between the EEG index of learning upon awakening and the percentage of trials containing slow waves during exposure (Fig. 6b; *r*(18) = −0.49, *P* = 0.030). When mapping this correlation onto scalp sensors, the effect was localized on frontal electrodes, where slow waves are generally most prevalent^44^, and also on central electrodes, where the effect of learning was observed in wakefulness (Fig. 2b and Supplementary Fig. 3).

**Fig. 7:**
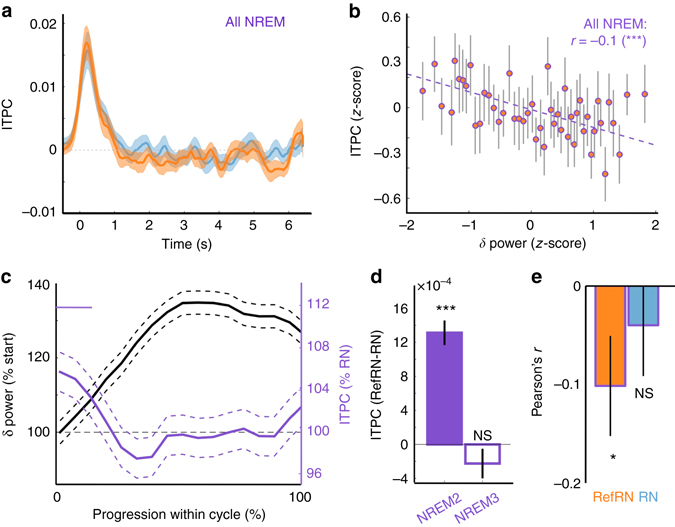
The learning index is dynamically correlated with slow-wave power in NREM sleep. **a** Inter-trial phase coherence (ITPC) extracted around 2 Hz ([1.5, 3.5]Hz) locked to stimulus onset for RefRN (*orange curve*) and RN (*blue curve*) trials in NREM sleep (NREM2 and NREM3). ITPC was corrected by the baseline ITPC ([−1.3, 0.3]s). **b** Correlation between ITPC values computed for RefRN trials (*z*-scored per sleep-cycle to highlight the within-cycle dynamics, see Methods) and *δ* (<5 Hz) power (*N* = 1368 data points in 82 cycles and 18 participants). Data was binned for illustrative purpose (*N* = 50 bins) and each dot represents a bin. Error bars represent the standard error of the mean (SEM) of ITPC values for each bin. Mixed-effects models revealed a significant influence of *δ* -power on ITPC (see Methods) quantified here with a Pearson’s correlation coefficient (***P < 0.001) as well as the regression line between the two variables, both estimated on unbinned data. **c** Evolution of longer-term memory index (ITPC, RefRN over RN ratio) and *δ* -power within sleep cycles. Cycle durations were normalized and expressed as a percentage of total duration. *δ* -power was also normalized (100% = beginning of cycle). *Dotted curves* denote the SEM across sleep cycles (*N* = 82 in 18 participants). Note the perceptual learning (RefRN>RN, *P*_cluster_<0.05) at the beginning of sleep cycles (typically light NREM). This advantage disappeared with the increase in *δ* -power. **d** Difference in ITPC between RefRN and RN (longer-term memory index) in NREM2 and NREM3 (all sleep recordings aggregated: *N* = 3698 and 2480 data points for NREM2 and NREM3, respectively, in 20 participants). The number of data points being different for RefRN and RN trials, the mean ITPC value for RN trials was subtracted to the ITPC for RefRN (unpaired subtraction). Mixed-effects models revealed a significant interaction between sleep stages and stimulus condition (*χ* ^2^(5) = 432.0, *P* < 2.2 × 10^−16^, see Methods). *Stars* show *post hoc* statistical tests comparing ITPC for RefRN and RN trials in NREM2 (two-tailed *t*-tests: ***P < 0.001) and NREM3 (NS: *P*≥0.05). **e** Pearson’s correlation coefficients between ITPC and *δ* -power for RefRN (*orange*) and RN (*blue*), respectively, computed on each cycle separately and averaged here across sleep cycles (*n* = 82 cycles). Pearson’s coefficients were significantly negative for RefRN (two-tailed *t*-test, *t*(81) = −2.24, *P* = 0.028, Hedges’ *g* = 0.25) but not for RN trials (*t*(81 = −0.50, *P* = 0.62, Hedges’ *g* = 0.06)

The suppressive effect of deep NREM was further linked to the predominance of slow waves during this phase of sleep^38^. Although there was no significant difference in ITPC between RefRN and RN trials when considering all NREM trials (NREM2 and NREM3, Fig. 7a), mixed-effects models (see Methods) revealed a highly significant influence of the power in the *δ* band (<5 Hz. a good proxy for slow wave density^45^) on ITPC values for RefRN trials (comparison with a model not taking into account *δ*–power to predict ITPC: *χ* ^2^(1) = 19.18, *P* = 2.2 × 10^−5^). Strikingly, this effect was restricted to RefRN and was not present for RN trials (*χ* ^2^(1) = 0.94, *P* = 0.33). We later quantified this relationship using a Pearson’s correlation and found a clear negative correlation between *δ* power and ITPC for RefRN trials (Fig. 7b, *r* (1392) = −0.12, *P* = 1.2 × 10^−5^) but not for RN trials (*r*(1392) = −0.026, *P* = 0.33).

We further examined whether the negative correlation between ITPC and *δ* -power over the entire night was maintained within individual sleep cycles. To do so, we normalized the sleep cycles’ durations (see Methods, *N* = 82 cycles in 18 participants; 2 participants did not have clearly identifiable sleep cycles) and examined the time course of *δ* power and ITPC. A clear increase in *δ* power was visible within the cycle progression, corresponding to the transition from lighter stages of NREM to deep NREM3 (Fig. 7c). At the beginning of the cycles, higher ITPC were observed for RefRN compared to RN (*P*_cluster_ < 0.05), consistent with learning occurring during light NREM sleep (Figs. 3 and 4). Later in the sleep cycle, the advantage for RefRN decreased and was eventually canceled out, closely mirroring an increase in *δ* power. Correlations were also observed between EEG markers of learning and other features of slow waves (density, slope, spatial expanse, and number of negative peaks), likewise modulated across sleep cycles (Supplementary Fig. 9). The observation of an effect of learning followed by its suppression over the time span of a sleep cycle could explain why we observed markers of learning in NREM2 but not in NREM3 (Fig. 7d) or when considering all NREM trials together (Fig. 7a). Finally, the negative correlation between ITPC and *δ* power was also observed within cycles for RefRN but not for RN trials (Fig. 7e). However, this reversed modulation of ITPC was rather small in proportion compared to the modulation of *δ* power (one order of magnitude smaller).

## Discussion

Using an auditory perceptual learning task, we tracked the formation and longer-term maintenance of memory traces from sleep to wakefulness. First, we demonstrated that new representations of complex acoustic stimuli could be formed during sleep. Second, behavioral performance upon awakening and neurophysiological markers extracted during sleep revealed a sharp distinction between REM and light NREM sleep on the one hand and deep NREM sleep on the other: while REM and light NREM sleep induced learning, deep NREM sleep suppressed learning. These learning and suppression effects transferred to wakefulness. A more detailed examination of EEG markers during the night further specified the electrophysiological activity that favored or, in contrast, suppressed learning. For REM sleep, perceptual learning was primarily driven by tonic REM. In light (NREM2) sleep, perceptual learning was correlated with the density of slow spindles. In deep (NREM3) sleep, the suppressive effect may be driven by the presence of slow waves. These findings could provide new insights into the function of sleep and its distinctive phases.

First, we reported that tonic episodes of REM sleep promote the learning of novel information in a way that is comparable to wakefulness. Just as during wakefulness, repeated exposure to noises during REM sleep leads to subsequent improvement in behavioral performance, along with corresponding neurophysiological markers of learning^30^. We observed evoked potentials (MEPs) for recurrently presented noise snippets as well as an increase in phase coherence across trials, demonstrating sleepers’ ability to form new representations for initially nondescript noises. Although the MEPs observed in REM sleep displayed a different shape compared to wakefulness, they were comparable to the typical auditory-evoked potentials observed in REM sleep^36^. Recurrent exposure (RefRN) enhanced specifically the P2 potential, as is the case for perceptual learning in wakefulness^46^. We also observed an increase in *θ* power for learnt trials (RefRN) in REM sleep, which is reminiscent of the enhancement of *θ* oscillations during the encoding of new memories^47^.

That listeners may learn novel sensory information during sleep is broadly consistent with previous research showing that sensory processing and the learning of new associations are possible during REM sleep^7, 28^. However, this also seems at odds with the relatively low incorporation of external stimulations to the dream scenery, classically interpreted as the brain shutting down from external inputs^37^. To reconcile these observations, it is worth noting that the memory effect was positively associated with the proportion of tREM, and thus negatively correlated with the proportion of pREM. This result is in line with previous findings of increased sensitivity to sensory information in tREM^39, 48^. Interestingly, dream reports seem more vivid in pREM^49^. It is therefore possible that, in pREM, the brain is more isolated from sensory input due to the processing of dream contents^37^. On the contrary, tREM would be more permeable to external sensory information. Thus, in REM sleep, the bottleneck limiting learning would be the connectedness to the environment^50^. However, further investigations are needed to clarify the respective influence of pREM and tREM on sensory processing and memory formation.

In the past, the observation of faithful sensory encoding^51–53^ and/or complex information processing in NREM sleep^25, 26, 28, 54, 55^ challenged the notion of a thalamic gating^18^ and the idea that sleepers do not have access to information from their environment in NREM sleep. Here, we extended these findings by providing compelling evidence for the learning of complex and novel sensory information during NREM2 sleep (i.e., light NREM sleep) through the presence of EEG markers of perceptual learning. Such a form of learning may involve a network of brain regions comprising secondary auditory cortices and the hippocampus^56^, suggesting again that a rather extensive network can be recruited and coordinated in NREM sleep. However, we do not provide evidence here that hippocampal structures were actually involved in the learning observed during sleep. The hippocampus rather could be involved in the formation of episodic memories for the learnt noise patterns, which could be restricted to wakefulness, while in sleep, only hippocampal-independent and implicit form of learning could occur.

Such preservation of complex information-processing abilities could be supported by local modulations of sleep depth, a phenomenon called local sleep. Indeed, sleep is not a monolithic phenomenon but that different brain regions can show different activity patterns^57^. In particular, some brain regions can recover wake-like activity in the absence of awakening at the scalp level^58^. Interestingly, in NREM2, recurrently presented noises were accompanied by the presence of evoked potentials resembling wake responses. This contrasts with the evoked potentials observed in NREM3 and classical auditory-evoked potentials reported for NREM sleep. Thus, the presence of wake-like activity in NREM2 could be interpreted as the recovery of some local wake-like processing in the context of global NREM sleep. The region-specific decrease in the magnitude of NREM sleep oscillations further strengthens this interpretation. Overall, our data argues that sleep depth may be flexibly modulated depending on sensory input, to allow for the timely recovery of wake-like information processing and learning.

In deep NREM sleep, however, memories seemed to be suppressed. Indeed, exposure to novel information in NREM sleep not only failed to improve participants’ performance upon awakening, but, rather, impaired their ability to learn this exact same information when awake. In addition, while EEG markers of learning were readily observed in light NREM2 sleep, they were markedly absent in deep NREM3 sleep. Such a contrast between light NREM2 and deep NREM3 sleep is consistent with a qualitative distinction between these two sleep stages in relation to neural plasticity^22^. According to this view, light NREM2 sleep favors synaptic potentiation, while deep NREM3 sleep favors synaptic downscaling.

Could sleep rhythms such as slow oscillations and sleep spindles fully account for this light vs. deep NREM dissociation? In our study, frontal sleep spindles were positively correlated with the positive learning effect. Accordingly, numerous studies have linked sleep spindles and memory consolidation^13^. In addition, *in vitro* studies showed that neuronal activations mimicking sleep spindles could induce long-term potentiation and could therefore represent a temporal window in which new synaptic contacts are created or reinforced^59^. We also found that slow waves were negatively correlated with memory formation. This is consistent with the idea that slow waves could trigger long-term depression^60^. However, sleep spindles are typically nested within slower oscillations, which argue against diametrically opposed roles^61^. In addition, the effect of spindles on plasticity also depends on the presence of slow oscillations^59^. Finally, both rhythms are present in light and deep NREM sleep and therefore cannot fully explain the differential impact of these sleep stages on learning.

We thus propose that a contextual change from light to deep NREM sleep explains the observed contrast. Indeed, sleep spindles and slow oscillations are known to undergo transformations from light to deep NREM sleep^62^. In particular, slow oscillations decrease in amplitude, slope, spatial expanse, but increase in density. Two types of synchronization processes have been proposed to account for these changes. In light NREM sleep, subcortico-cortical processes, potentially stimulus-driven, would occur (type-I slow waves, corresponding approximately to K complexes), leading to high-amplitude, steep and widespread slow waves. These type-I waves could be associated to activations of the arousal system, restoring some ability to process sensory information and to form new memory traces. In deep NREM sleep, however, slow waves (type-II) would arise from local cortico-cortical synchronization processes, and could subserve the deep NREM suppressive effect observed. Accordingly, the suppression effect was paralleled with the emergence of more numerous but more local, and putatively cortico-cortical, slow waves. Nonetheless, further investigations are needed to prove the relationship between deep NREM sleep slow waves and the suppression of memories, as our interpretation is mostly based on correlational analyses.

Understanding why both REM sleep and light NREM sleep favor learning while deep NREM sleep suppresses it could provide a unified view of the impact of sleep on memory formation. Changes in the level of neuromodulators across sleep phases could be responsible for such a reversal. In particular, Acetylcholine (ACh) drops in slow-wave sleep compared to both wakefulness and REM sleep^63^. Interestingly, ACh can control the polarity of spike-timing-dependent plasticity (STDP^64, 65^). This is relevant to our experimental results, as modeling work showed that STDP is sufficient to form a memory trace from recurrent random inputs^66^. Thus, an ACh-dependent modification of the STDP rule could tentatively account for our results. Under higher levels of ACh (wakefulness, REM sleep, and perhaps in some specific parts of light NREM sleep), learning would occur through the potentiation of the synapses recruited by the recurrent acoustic signal^66^. Under low levels of ACh (deep NREM sleep), the same synapses would, on the contrary, be downscaled, so not only would learning not occur, but the subsequent reactivation of the specific network recruited by a noise sample would also be suppressed upon wakefulness. It has been proposed that such a downscaling process would, in fine, participate to sleep-related memory consolidation^12, 21, 67^. We do not suggest any functional role for the suppressive effect of information presented during sleep; rather, it seems an inevitable byproduct of the synaptic downscaling needed for homeostatic purposes^12^.

But how can synaptic downscaling explain the suppression of learning upon awakening? An initial hypothesis is that the recurrent presentation of a RefRN stimulus during sleep changed the initial conditions of the network involved in learning in such a way as to make it harder to learn that particular exemplar of noise. Indeed, in wakefulness not all RefRN are learnt equally easily by all listeners^29^. This has been explained by idiosyncratic learning of local patterns within the noise^30^. From a mechanistic point of view, such local patterns could activate a pre-existing network of neurons within the auditory cortices, and when the pattern is repeated, the corresponding network may be strengthened. Which noises possess local patterns able to bootstrap the process depend on the initial state of the network. However, because of synaptic downscaling, the activation of the pre-existing network during NREM sleep could also lead to its degradation rather than its potentiation, preventing the initiation of the bootstrapping of learning upon awakening. Thus, not only would a RefRN target presented during synaptic downscaling phases not be learnt, but such targets would be even harder to learn (compared to new stimuli) upon awakening. An alternative explanation is that RefRNs were learnt during NREM sleep, but the corresponding mnesic traces were suppressed or harder to recruit upon awakening. The suppression of learning after exposure in NREM sleep is unprecedented, and future studies will help clarifying the exact mechanisms enabling memory suppression.

Finally, the question of how our results generalize to other forms of learning or synaptic plasticity remains to be further investigated. Indeed, the mechanisms underlying the perceptual learning of acoustic noise are still unclear. Previous studies showed an involvement of hippocampal structures^56^ as well as the formation of new auditory objects^30^, which suggests a parallel with more classical forms of hippocampal-dependent memories. The fact that such learning can last for several days lends evidence to this argument^29^ and makes it unlikely that such form of learning reflects a mere and short-lived adaptation effect. Nonetheless, there is to date no evidence demonstrating that the hippocampus is necessary for this form of learning. A simple interpretation of noise learning is that it recruits core neuroplastic processes such as STDP, which are present at many stages of cortical processing. While this view is supported by computational models^66, 68^ and large-scale recordings^30^, direct empirical evidence at the synaptic level is still missing.

## Methods

### Participants

Twenty right-handed subjects (11 females, age 20–31 years) with no history of neurological or sleep disorders participated in this study. They filled in questionnaires about their sleep habits and had an interview with a sleep specialist prior to recordings. Sleep habits matched the general population standards. Participants were monitored for 7–10 days prior to the recording session through actigraphy and sleep diaries to ensure stable sleep/wake rhythms. The sample size was determined based on previous studies on (i) sensory processing during full-night polysomnographic recordings^50^ and (ii) learning of acoustic noise using electroencephalographic (EEG) recordings^30^. This protocol was approved by the local ethics committee (Comité de Protection des Personnes, Ile-de-France I, Paris, France).

### Sleep Study and Noise-Memory Paradigm

On the day of the recordings, participants were first familiarized with the stimuli used in our protocol (white-noise acoustic stimuli). They were equipped for polysomnographic recordings and performed an initial pre-sleep phase while remaining awake (Fig. 1c, 41 ± 1 min, mean ± standard error of the mean (SEM) across participants) consisting in the detection of repetitions in acoustic noise (see below). They went then to bed and were asked to perform the same task as long as they were awake. Stimuli were continuously presented over the whole night (sleep phase: 494 ± 20 min). Finally, upon awakening, participants underwent a memory test (post-sleep phase) without being explicitly told so; i.e., they were instructed to keep on detecting repetitions within noise trials (89 ± 2 min). Polysomnographic equipments were removed at the end of the post-sleep phase.

We used a variant of the noise-memory paradigm (Fig. 1b)^29^, which had been optimized for Electroencephalographic (EEG) recordings^30^. Recording sessions were preceded by a short familiarization phase during which we played sounds with or without repetitions to participants while indicating to them which stimuli included or not repeated patterns. Then, each recording session was separated in three different phases (Fig. 1c). In the initial pre-sleep phase, participants were instructed to discriminate the following: (i) noise stimuli (N, duration: 3.5 s), i.e., acoustic stimuli made of ever-changing white noise and thus deprived of any repeating sequence, (ii) repeated-noise (RN) stimuli in which a 0.2 s white-noise target was presented 5 times to listeners (Fig. 1b). In RN trials, the noise targets were interleaved with ever-changing white-noise fillers to keep stimulus duration similar to N trials (3.5 s). The first target was presented 0.8 s after stimulus onset and targets were presented every 0.5 s. Both target and fillers being made of white noise (no sample-to-sample predictability), such concatenation is seamless as illustrated in Supplementary Fig. 2 (no change in sound envelope for example). Repeated noise targets differed from one trial to the other. In the RN condition, we thus introduced a repetition of the same piece of acoustic information within but not across trials (Fig. 1b). A different set of RN targets was presented to each participant (Fig. 1a). Unknown to participants, another set of repeated targets (*N* = 5 for each participant) was randomly selected to be recurrently presented across the entire pre-sleep phase. Such trials were termed RefRN stimuli and correspond to the presentation of the same targets both within and across trials. Classically, RefRN trials are associated with improved repetition-detection performance compared to RN trials^29–31, 69^. From the perspective of participants, RefRN and RN trials differed only through prior exposure as they shared the same structure. Thus, the difference in performance between RefRN and RN trials can be used to titrate longer-term perceptual learning. The ability to differentiate RN trials from N trials on the other hand may involve the rapid formation of memory to noise^30^ (Fig. 1a). We provide two audio exemplars of N (Supplementary Audio 1) and RN/RefRN (Supplementary Audio 2) stimuli.

Lastly, a fourth type of stimuli (Reference Noise, RefN) was introduced to balance the number of trials with and without repeating patterns. In these trials, the 0.3-s-long noise snippets used to build RefRN trials were used and injected every 0.5 s. However, contrary to RefRN, we used different RefRN targets to build a single RefN. Thus, there was no within-trial repetition of a target in RefN trials but RefN trials did contain fragments that were previously played to participants (RefRN targets) and potentially learnt. Our expectation was that RefN trials would probe participants to wrongly indicate the presence of repetitions due to the presence of known fragments. However, these RefN trials did not differ from N trials in the pre-sleep phase, neither regarding behavior nor EEG recordings, and thus they were not further analyzed.

Response handles were attached to participants’ hands, who were instructed to indicate the presence of a repeating pattern by pressing the right or left handle (the ‘response-side/stimulus-condition’ mapping was counterbalanced across subjects). Response-side and reaction times (RTs) were recorded for further analysis. Participants were instructed to remain awake and to respond to stimuli during the entire pre-sleep phase while remaining eyes-closed. Stimuli were played every 5.5 to 7.5 s (jitter: uniform distribution) with a break every 64 trials.

In the sleep phase, similar stimuli were used. N and RN stimuli were freshly generated for each N or RN trial. However, different sets of RefRN targets were played in periods of wakefulness (same as the pre-sleep phase), NREM (*N* = 5 NREM RefRNs), and REM sleep (*N* = 5 REM RefRNs) according to an online assessment of vigilance states (Fig. 1c). In practice, when participants were awake, only the RefRN containing the wake targets were played (wake RefRN). In NREM sleep (NREM2 and 3), the NREM set of RefRN was played to participants, and, in REM sleep, the REM set was used. Each time participants awoke, the RefRN list was set back to wake RefRN targets. In addition, when the NREM or REM RefRN sets were played, the duration of stimuli was increased (6 s instead of 3.5 s in wakefulness) in order to double (10 vs. 5) the number of within-trial repetitions in RefRN and RN trials. Yet, the general structure (0.2 s-long targets separated by 0.3s-long white-noise fillers) was conserved. Participants were instructed to respond to stimuli as long as they would remain awake and to resume responding in case of an awakening. They were verbally remembered to do so by the experimenter, in case of prolonged awakening without responses (no response while participants were awake and stimuli were being played for about 5 min). Stimuli were played every 6.5–9.5 s in wakefulness and every 9–12 s in sleep (jitter: uniform distribution).

Finally, in the post-sleep phase, participants were tested on all RefRN targets presented in the pre-sleep and sleep phases (*N* = 5 wake, NREM and REM RefRN targets per participant) along 5 new RefRN targets. Task instructions remained the same (detection of repetitions in noise) and participants were not informed of the presence of previously presented noises. Each RefRN was tested in a separate 5-minute block along freshly generated RN and N trials and was presented 8 times. The order of presentations of wake, NREM, REM, and new RefRN was randomized. Stimuli were played every 5.5–7.5 s (jitter: uniform distribution). Participants were instructed to remain awake and to respond to stimuli in the entire post-sleep phase. However, in some cases, participants failed to indicate the presence or absence of repetitions. Post-sleep blocks with more than 20% trials without responses were excluded from our analysis (17 out of 400 blocks in 20 participants). Participants never received feedback on their response in the pre-sleep, sleep and post-sleep phases.

All stimuli were randomly generated to create acoustic white noise (sampled at 44,100 Hz). Each stimulus is therefore made of thousands of normally distributed numbers. White-noise stimuli have a flat spectrum on average, constant amplitude envelope, and are deprived of short-term regularities (no sample-to-sample predictability) or salient features making the detection of any pattern very difficult (Supplementary Fig. 2). In addition, as stimuli were randomly generated, prior exposure could be precisely controlled as the probability, for each participant, to have encountered the exact same noise segments before the experiment is close to 0. The white-noise learning paradigms provide therefore a unique opportunity to investigate the learning of novel sensory information. Stimuli were presented to participants using the PyschToolbox extension^70^ for Matlab (Mathworks Inc., Natick, MA, USA) and were played at 50 dB (soundcard: Echo Indigo, Echo Digital Sound Corp., Santa Barbara, CA, USA) through a loudspeaker placed near the bed to ensure comfortable listening conditions while minimally disturbing sleep.

### Contrasts of interest and expected results

As thoroughly discussed recently^30^, the noise-memory paradigm allows exploring the rapid formation of memory to noise at different time scales. The fact that listeners could discriminate between RN and N trials demonstrates their ability to detect the reoccurrence of a nondescript noise segment embedded in running noise after only few presentations (max: 5 in the pre-sleep phase) and despite the statistical similarity between targets and fillers. Therefore, the RN vs. N contrast reveals the formation of a form of shorter-term memory to noise (Fig. 1a, *right*). On the contrary, RefRN and RN stimuli have identical structures (Fig. 1b). They only differ through participants’ prior exposure. Improvement in repetition-detection performance for RefRN compared to RN trials can only be explained by the formation of longer-term memory to noise (time scales of minutes or hours; Fig. 1a, *right*). Such longer-term learning of acoustic noise has been confirmed by several studies^29–31, 69^. Importantly, Agus and colleagues showed that such learning was preserved after 2 weeks^29^. We thus used the RefRN vs. RN contrast to focus on longer-term memory (across-trial) while the RN vs. N trials were used to target shorter-term memory (within-trial; Fig. 1a). The RefRN vs. N contrast focuses on the cumulative effect of shorter- and longer-term memory.

### Electrophysiological recordings

Participants were equipped for polysomnographic recordings according to the ASSM guidelines^38^. We continuously recorded electroencephalographic (EEG, *N* = 19 derivations, 10–20 montage), electro-oculographic (EOG, *N* = 2 derivations, placed above and under the right and left canthus, respectively), electromyograhpic (EMG, one derivation on the chin and two derivations on right and left abductor pollicis brevis (thumb flexor muscle) recording muscle activity associated to hand responses), and electrocardiographic (ECG, *N* = 1 derivation) data in parallel with video monitoring. To ensure the reliability of data collection through hours of recordings, AgCl electrodes were attached to participants’ scalp using an adhesive paste (EC2, Natus Neurology Inc., Middleton, WI, USA). This technique, while minimizing electrodes’ displacement, limits the number of channels that can be recorded. Electric signals were amplified through a B1IP or B2IP MEDATEC amplifier (Medical Data Technology SPRL, Bruxelles, Belgium). The signal corresponding to the EEG and EOG channels was recorded as the difference in voltage between each sensor and a ground electrode placed on participants’ scalp, near the vertex (i.e., near Cz). EEG electrodes were re-referenced offline to the averaged mastoids, and EOG electrodes were re-referenced to the opposite mastoids. During recordings, both EEG and EMG were re-referenced to the opposite mastoids. EMG consisted in bipolar derivations with two recording electrodes placed few centimeters apart on participants’ skin. EEG, EOG, ECG, and EMG data was recorded at a 200 Hz sampling rate. Impedances of scalp electrodes were generally below 5kΩ. An external channel was used to synchronize EEG data with stimuli presentation times.

Participants were constantly monitored during both wakefulness and sleep. As explained above, during the sleep phase, a given set of RefRN (wake, NREM, or REM) was selected according to participant’s vigilance state. To do so, the vigilance state was assessed online using standard guidelines^38^ by an experienced scorer (TA) and confirmed offline by two scorers (TA and DL) blinded to experimental conditions (see below and Supplementary Table 1).

### Behavioral indices of perceptual learning

To behaviorally assess listeners’ ability to detect the presence of repeating noise segments, we computed their sensitivity to the presence of these repetitions by means of a *d*′ index^71^. The *d*′ index has the advantage to take into consideration participants’ biases for one response (presence of repetitions) or the other (absence), facilitating the averaging across participants. A significant deviation of the *d*′ from 0 indicates participants’ ability to reliably discriminate the two conditions of interest at the group level. *d*′ indexes were computed for RefRN and RN conditions independently and for each participant (see Eqs. 1 and 2):

1 $$d_{{\rm{Re}}\,{\rm{fRN}}}^\prime = z\left( {{\rm{Hi}}{{\rm{t}}_{{\rm{Re}}\,{\rm{fRN}}}}} \right) - z\left( {{\rm{F}}{{\rm{A}}_{\rm{N}}}} \right)$$

2 $$d_{{\rm{RN}}}^\prime = z\left( {{\rm{Hi}}{{\rm{t}}_{{\rm{RN}}}}} \right) - z\left( {{\rm{F}}{{\rm{A}}_{\rm{N}}}} \right)$$

where *z*(*x*) corresponds to the *z*-score for proportion *x*, Hit_RefRN_ corresponds to the proportion of correct responses for RefRN trials, Hit_RN_ corresponds to the proportion of correct responses for RN trials, and FA_N_ corresponds to the proportion of incorrect responses for N trials. Extreme performances (100%/0%) were adjusted to the equivalent of half of a single correct/incorrect response^71^ to avoid infinite *d*′ values. As previously shown, RefRN trials were associated to higher *d*′ indexes compared to RN trials (Fig. 2a, *top*).

Reaction times (RTs) also capture the formation of memory traces to noise^30^. Typically, RefRN trials lead to faster responses, often anticipating the end of the stimulus presentation window (<3.5 s; Fig. 2a, *middle*). We therefore combined the improvement in response accuracy and rapidity to titrate the amount of learning. To do so, we used the Behavioral Efficacy (BE) index, which we used in a similar experimental context^30^. Inspired by the Inverse Efficiency Score^72^, BE was defined as follows:

3 $${\rm{B}}{{\rm{E}}_{{\rm{Re}}\,{\rm{fRN}}}} = d_{{\rm{Re}}\,{\rm{fRN}}}^\prime \times \left( {\frac{{{\rm{R}}{{\rm{T}}_{\rm{N}}}}}{{{\rm{R}}{{\rm{T}}_{{\rm{Re}}\,{\rm{fRN}}}}}}} \right)$$

4 $${\rm{B}}{{\rm{E}}_{{\rm{RN}}}} = d_{{\rm{RN}}}^\prime \times \left( {\frac{{{\rm{R}}{{\rm{T}}_{\rm{N}}}}}{{{\rm{R}}{{\rm{T}}_{{\rm{RN}}}}}}} \right)$$

where RTs for RefRN and RN trials were computed from stimuli onsets. Intuitively, BE is increased for high *d*′, and if the RTs to the stimulus of interest were faster than the N baseline. BE was higher for RefRN trials compared to RN trials (Fig. 2a, *bottom*).

Behavioral data was analyzed in the pre-sleep (Fig. 2a) and post-sleep (Fig. 3) phases but not in the sleep phases due to the absence of behavioral response during sleep. Trials without responses were not included in behavioral analyses. In the sleep phase, RefRN targets were presented according to participants’ vigilance state. However, in the course of the night, some of these RefRN have been presented around microawakenings, as assessed by a double offline scoring (*N* = 38 over 100 RefRN targets in NREM sleep and 18 over 100 in REM sleep). However, the isolated presentation of NREM targets during wakefulness can hardly explain the suppressive effects observed for NREM targets. Nevertheless, in the post-sleep phase, to avoid this confound and to make sure that the positive effect for REM targets could not be due to these awakenings, BE was computed when excluding all NREM or REM RefRN heard around (micro)-awakenings (Supplementary Fig. 4c), which led to identical results as in Fig. 3.

### Offline sleep scoring of polysomnographic recordings

Polysomnographic data was analyzed using a combination of SPM (Functional Imaging Laboratory, Univ. College London, London, UK), FieldTrip^73^, and EEGlab^74^ toolboxes running on Matlab (Mathworks Inc., Natick, MA, USA).

Polysomnographic data (EEG, EOG, EMG, and ECG data) was preprocessed according to established guidelines. EEG data was high-pass filtered above 0.1 Hz and then low-pass filtered below 30 Hz (5th order two-pass Butterworth filters). EMG was were band-pass filtered between 60 and 80 Hz (5th order two-pass Butterworth filter). In addition, EEG, EOG, EMG, and ECG were notch-filtered around 50 Hz to reduce line noise. Vigilance states were assessed online using standard guidelines^38^ by an experienced scorer (TA) and confirmed offline on 20s-long windows by two scorers (TA and DL) blinded to experimental conditions. Polysomnographic was were continuously scored on 20-s-long windows as follows: wakefulness, NREM sleep stage 1 (N1), NREM sleep stage 2 (N2), NREM sleep stage 3 (N3), tonic REM sleep (tREM), and phasic REM sleep (pREM). The NREM sleep stages were here labeled as NREM1, NREM2, and NREM3 to avoid confusions with the ERP nomenclature. Only Fz, C3, C4, and Pz EEG derivations from the classical 10–20 montage were used for scoring. The disappearance of the rhythms associated to wakefulness such as alpha oscillations ([8–10] Hz) and the apparition of slow rolling eye movements were indicative of the transition to NREM1. NREM sleep hallmarks (K complexes and sleep spindles) marked the transition to deeper stages of NREM sleep (NREM2 and NREM3). REM sleep was characterized by the recovery of an EEG signal similar to wakefulness coupled with a highly reduced EMG and the occasional presence of rapid eye movements (REMs) performed with eyelids closed. Epochs of REM sleep containing at least one REM were scored as phasic REM sleep while epochs of REM sleep without any REM were scored as tonic REM sleep. In addition, epochs showing signs of arousal (body movements, increase in alpha oscillations, or oscillations above 16 Hz) in association with trial onsets were marked, and the corresponding trials were not included in the sleep analyses.

Supplementary Fig. 1 shows representative examples of these different sleep stages and Supplementary Table 1 summarizes sleep scoring across participants. In addition, the spectral profiles of sleep stages were in accordance with the literature (Supplementary Fig. 7b, c). Finally, in NREM sleep, slow waves and sleep spindles were detected using automated algorithms to perform quantitative analyses on the influence of these sleep patterns (see below). Spatial distributions of average densities are shown in Supplementary Fig. 8, which are again in accordance with the literature^44, 62^.

As the offline scoring was performed post hoc, in some cases the scoring of a given trial did not correspond to the RefRN list that was played at that time. This may be due to errors during the online assessment of vigilance states or to the participant suddenly transitioning to a different sleep stage. To avoid potential confounds, the offline scoring was used as a reference and the corresponding trials were discarded from the analyses of sleep recordings. On average, for the NREM list, this happened 2.5 ± 0.6 times (mean ± SEM) in wakefulness, 4.7 ± 0.8 in NREM1, and 5.5 ± 1.1 times in REM sleep (compared to 207.6 ± 10.0 RefRN trials on average in NREM2, and 144.7 ± 9.1 in NREM3). For the REM list, this happened 1.1 ± 0.3 times in wakefulness, 3.3 ± 0.9 in NREM1, 3.5 ± 0.9 times in NREM2, and never in NREM3 (compared to 80.8 ± 7.4 RefRN trials on average in REM sleep).

### Identification and detection of sleep cycles and rhythms

Sleep cycles were individualized using participants’ hypnograms (97 cycles in 18 participants, 5.6 ± 0.2 per participant, mean ± SEM). In sleep cycles having different durations (86 ± 3.6 min), we normalized cycles’ length to be able to average variables of interest across cycles (*N* = 18 bins). The progression within the cycles was therefore expressed in percentage of the total duration (Fig. 7; Supplementary Fig. 9). Eighty-two (82) cycles in 18 participants were eventually included in the analysis, the others not having enough RefRN or RN trials (at least 20 trials per condition and per bin, see below). Two participants were not included in the sleep-cycle analysis due to the difficulty of clearly identifying sleep cycles.

Slow waves and sleep spindles were detected in NREM sleep using algorithms that have been presented in details elsewhere^75, 76^. For each slow wave, we extracted its onset, peak-to-peak amplitude (amplitude), down-to-up state slope (slope), number of negative peaks, and spatial expanse (i.e., for a given channel of reference, here Cz, and for each slow wave, the proportion of channels also showing a slow wave in a 100 ms window centered on the reference slow wave’s starting point). For each spindle, we computed its frequency by extracting the peak in power (estimated through a Fast-Fourier Transform, FFT) within a [11, 16] Hz window. Spindles with a frequency below 13 Hz were declared slow spindles and spindles with a frequency above 13 Hz, fast spindles^75^. Scalp distributions of the densities of detected events are shown in Supplementary Fig. 9. It is worth noting that the slow-wave detection well-replicated recent findings on the changes in slow-wave properties from light to deep NREM^62^. In particular, the density of slow waves and the number of negative peaks robustly increased during sleep cycles while their slope or spatial expanse decreased (Supplementary Fig. 9). As for the spindle detection, it replicated the known frontal distribution of slow spindles and centro parietal distribution of fast spindles^77^.

In order to compute the percentage of trials associated with slow waves, fast, and slow sleep spindles (Fig. 6b), we examined, for each trial in NREM2 and 3 stages, whether a slow wave or fast or slow sleep spindle was detected during the presentation of the stimulus. The channel used corresponded to the electrode with the highest density for the corresponding graphoelement (slow waves and slow spindles: Fz; fast spindles: Pz, green dot in Supplementary Fig. 8).

### Electrophysiological Indexes of Perceptual Learning

Electrophysiological (EEG) data was first high-pass filtered above 0.1 Hz (5th order two-pass Butterworth filter) and then epoched on large temporal windows ([−14, 14] s) around stimulus onsets. EEG was were then low-pass filtered below 20 Hz (5th order two-pass Butterworth filter), and a notch-filter at 50 Hz was also applied to reduce line noise. A second epoching on shorter windows was performed ([−2, 7] s). Data was corrected for baseline activity after each epoching by subtracting prestimulus activity for each EEG derivation. Minimal artifact rejection was applied for the ERP (Figs. 2 and 4) and power analyses (Fig. 5) trials for which the maximal absolute value of the EEG signal in at least one of the central electrodes (C3, C4, and Cz) was higher than a given threshold (500 μV) were excluded from our analyses. We set here a high threshold to prevent discarding high-amplitude slow oscillations (slow waves, K complexes) as artifacts. Muscular artifacts were not corrected for by other means. It is worth noting that, in the sleep analyses, muscular activity and movements minimally impacted the EEG recordings as trials associated with arousals were marked and discarded during the online scoring. On average, 0.59 ± 0.34% of epochs were removed in NREM2, 0.84 ± 0.48% in NREM3, and 0.65 ± 0.67% of epochs in REM sleep (mean ± SEM across 20 participants). In this study, we focused mainly on central electrodes (C3, C4, and Cz in the 10–20 montage) as these electrodes show the largest responses to sounds^35^ and noise repetition^30^. All analyses were performed on these electrodes except when stated otherwise. When analyzing the data from C3, C4, and Cz altogether, MEPs, spectral power or ITPC were computed on each channel independently. The results of these analyses were then averaged across channels for each participant. The corresponding statistical analyses and plots show therefore brain activity averaged across central electrodes.

We focused on either stimulus-locked event-related potentials (ERPs; Supplementary Fig. 7) or target-locked MEPs (memory-evoked potentials: Figs. 2, 4). Stimulus-locked ERPs show EEG potentials triggered by the transition from silence to noise irrespective of experimental conditions. Indeed, we here focused on the late auditory-evoked potentials (AEPs^35^) occurring within the first 500 ms following stimulus onset and therefore before any presentation of a RefRN or RN target (starting at 800 ms). As classically observed, these AEPs present stereotypical and state-dependent profiles^35^.

We also computed target-locked MEPs. For target-locked MEPs, the EEG signal was high-pass filtered above 1 Hz instead of 0.1 Hz to get rid of slow drifts (as in ref. ^30^). These MEPs had the particularity to be computed within the stimulus presentation window. White noise being deprived of significant fluctuations in acoustic energy or salient perceptual landmarks that usually trigger ERPs (e.g., silence-to-noise transition in the case of AEPs), any deviation from the N condition for RefRN and RN trials can be interpreted as an indication that the brain had detected the presence of the repeated noise segment. We termed the ERPs associated to repeated targets’ Memory-Evoked Potentials (MEPs) to emphasize the fact that they parallel perceptual learning^30^. Comparing AEPs and MEPs can provide means to explore the neural mechanisms underlying MEPs and in particular whether they share common generators. Target-locked MEPs were first averaged from the 2nd to the last target (wake trials: 4 targets per trial; sleep trials: 9) for each trial and then averaged across trials for each participant and condition. A baseline correction (baseline: [−0.1, 0]s before target onset) was applied to each target.

Time–frequency decompositions were performed using the EEGlab toolbox^74^ on the EEG data epoched around stimulus onsets (Fig. 5). We employed the wavelet method. For a given scalp sensor, we obtained the decomposed signal *s*(*t,f*) for each time point (t) and frequency (f) in its complex representation:

5 $${S_{t,f}} = {A_{t,f}}{{\rm e}^{i\varphi_{t,f}}}$$

where *A*(*t,f*) reflects the amplitude of the EEG signal at a given frequency and time and *φ*(*t*,*f*) reflects its phase.

Power response for each condition and vigilance state was extracted from this time–frequency decomposition (Fig. 5). Power response was normalized by pre-stimulus onset activity ([−0.25, 0] s) and expressed on a log-scale as decibels.

Inter-trial phase coherency (ITPC) was also computed using wavelets. We focused on a frequency band ([1.5, 3.5] Hz) around stimulus presentation (2 Hz) based on previous studies^30, 31^ and the pre-sleep phase (Supplementary Fig. 3). ITPC describes how the phase of the EEG signal is reproducible across trials for a given condition and participant. Thus, high ITPC values across participants indicate that each participant exhibited a reproducible phase for the corresponding time and frequency (for a given condition), even if the particular phase differed between participants. To compute ITPC, we extracted the phase of the signal for each time and frequency (φ_t,f_) and averaged it across *n* trials using Euler’s formula:

6 $${\rm ITP}{{\rm C}_{t,f}} = \sqrt {\left( {\frac{1}{n}{{\left( {\mathop {\sum}\limits_n {\cos \left( {{\varphi _{t,f}}} \right)} } \right)\!\!}^2} + \frac{1}{n}{{\left( {\mathop {\sum}\limits_n {\sin \left( {{\varphi _{t,f}}} \right)} } \right)\!\!}^2}} \right)} $$

The presence of ERPs and the increase in ITPC are tightly linked: ERPs (and MEPs) lead to higher ITPC values as they have a reproducible shape across trials. We recently showed that the increase in ITPC associated to noise-learning could be explained by the presence of MEPs^30^. However, ITPC has several advantages compared to ERPs: (i) it allows targeting a certain frequency range; (ii) contrary to ERPs, it is not affected by high-amplitude physiological events (such as slow waves) or artifacts; (iii) it can capture non-time-locked activity (see ref. ^30^ for a comparison between ERPs and ITPC in the context of the noise-memory paradigm). We therefore focused on ITPC to compute an EEG index of perceptual learning (Figs. 4b, 6 and 7).

Such EEG index was particularly useful during sleep where behavioral responses are abolished, preventing the computation of any behavioral index of learning. Based on our previous work and on Supplementary Fig. 3a showing an increase in ITPC for RefRN and RN trials around 2 Hz in the pre-sleep phase, we extracted the average ITPC on a [1.5, 3.5] Hz window and during stimulus presentation (pre-sleep phase and memory test: [0.8, 3.8] s; sleep-phase: [0.8, 5.5] s). In the pre-sleep phase, the ITPC around 2 Hz was larger for RefRN compared to RN and correlated with behavioral performance (Fig. 2d). Thus, ITPC appeared here as a good proxy to assess the occurrence of perceptual learning and quantify it. ITPC was computed on C3, C4, and Cz channels separately and then averaged across these channels for each participant separately.

Lastly, as can be noted in Eq. 6, ITPC depends on the number *n* of trials on which it is computed. We kept this number identical across conditions: in the pre- and post-sleep phases, for each participant, the condition with the smallest number of trials was chosen as the reference and trials were randomly picked for the other, more numerous condition. During the night, ITPC was computed by dividing all sleep cycles into fixed windows of 20 stimuli presentations (either RefRN or RN). This was done either by cycle when focusing on the within-cycle dynamics (Fig. 7b-c) or when considering the entire night (Figs. 4b and 7d). These fixed windows were slid trial-by-trial. Thus, if a cycle (or a night) contained *n* RefRN trials, we obtained *n-19* ITPC values (that were then binned in 18 bins for each cycle, Fig. 7b). When examining the entire sleep recordings, we pooled data across participants. As the number of windows differed between RefRN and RN trials, statistical tests are unpaired in Fig. 4b and we subtracted the average ITPC for RN trials to the ITPC values for RefRN trials in Fig. 7d.

To obtain the power spectra displayed in Supplementary Fig. 7, we used a fast-fourier transform (FFT) and extracted the power for all trials altogether (and not per condition). We then averaged it in time across the entire epoch ([−2, 7]s).

### Statistics

Parametric statistics were used (Student *t*-tests to compare pairs of variables, Pearson’s method for correlations) when variables could be approximated to the normal distribution (Kolmogorov–Smirnov test). Otherwise, we used nonparametric statistics (Wilcoxon rank-test (*u*-test) to compare conditions and Spearman’s method for correlations) when data was not normally distributed. All tests applied here were two-tailed tests. When comparing two distributions or a distribution with a reference value, we estimated the effect size using Hedges’ *g* ^78^.

For Fig. 3c, two data points were detected as outliers when using an algorithm based on the ‘median absolute deviation’ method (see ref. ^43^ and the ‘robust correlation’ toolbox for Matlab). We therefore also reported the correlation coefficients when including these two data points in the Results section. However, as these correlation coefficients were not obtained when including all participants, they should be considered with caution.

For the null results illustrated in Fig. 3a, a nonparametric method (Bayes factors) was used to test the plausibility of the null hypothesis^34^. These Bayes factors are reported in the text. A Bayes factor comprised between 3 and 20 is usually considered as positive evidence for the null hypothesis, while a Bayes comprised between 20 and 150 reflects strong evidence for the null hypothesis^79^.

We also used a stepwise regression analysis (with forward selection) to examine the respective influence of NREM and REM sleep substages on the learning effects observed upon awakening. The aim was here to better assess the impact of sleep stages on perceptual learning while taking into account the fact that the amount of trials in these sleep stages are not independent from each other.

Statistics used for time and time–frequency plots were corrected for multiple comparisons by means of a cluster-permutation approach^80^. The rational is the following: each cluster was constituted by the samples (in a 1D (time plots) or 2D (time–frequency) space) that consecutively passed a specific threshold (here, *P* < 0.05 except for Fig. 7c where *P* < 0.01). The cluster statistics were chosen as the sum of the t-values of all the samples within the cluster. Then, we compared the cluster statistics of each cluster with the maximum cluster statistics of 1000 random permutations and obtained a nonparametric *P-*value (*P*_cluster_). Significant clusters are displayed as horizontal bars or contours on plots; *P*_cluster_ are reported in the text and figures’ legends.

When computing ITPC on small windows throughout the entire sleep recordings (Figs. 4b and 7d) or across sleep cycles (Fig. 7b), we used mixed-effect models to take into consideration the trial and subjectwise variances separately. Subject identity was considered as a random effect. Mixed-models analyses were performed in R (R Development Core Team) with the ‘lme4’ and ‘lmerTest’ R packages. In Figs 4b and 7d, we examined the influence of stimulus condition and sleep stages on ITPC values. We estimated the significance of the interactions between these two variables by comparing a model including only fixed effects vs. a model including fixed effects and their interaction. In Fig. 7b, to test the influence of *δ*-power on ITPC, we compared a model including *δ*-power as a predictor with a model considering only subject identity as a random factor. All model comparisons were performed with chi-square (*χ* ^2^) tests. The corresponding *χ* ^2^ and *P*-values are reported in the text.

### Data availability

All the relevant data is available upon reasonable request. Inquiries should be directed to the corresponding author.

## Electronic supplementary material

Supplementary Information

Supplementary Audio 1

Supplementary Audio 2
