# Supplementary material for: Formation and suppression of acoustic memories during human sleep
Source: Nat Commun. 2017 Aug 8;8:179. doi: 10.1038/s41467-017-00071-z (PMC5548898; doi:10.1038/s41467-017-00071-z)
Supplement: Supplementary file 1 — Supplementary Information [file 41467_2017_71_MOESM1_ESM.pdf]

File name: Supplementary Information
Description: Supplementary Figures and Supplementary Table

File name: Supplementary Audio 1
Description: Exemplar of a 3.5-s-long Noise (N) stimulus as described in Figure 1 and Supplementary Figure 2 as well as in the Methods section. N stimuli were made of acoustic white noise. No repeating pattern was injected in N stimuli.

File name: Supplementary Audio 2
Description: Exemplar of a 3.5-s-long Repeated Noise (RN) or Reference RN (RefRN) stimulus as described in Figure 1 and Supplementary Figure 2 as well as in the Methods section. RN and RefRN stimuli were also made of acoustic white noise. However, a 0.2-s-long segment of white noise was repeated 5 times here every 0.5s. Such repetition can be detected by human listeners, even more so after several exposures. High-quality sound devices (headphones or speakers) are preferable to detect the repeating pattern.

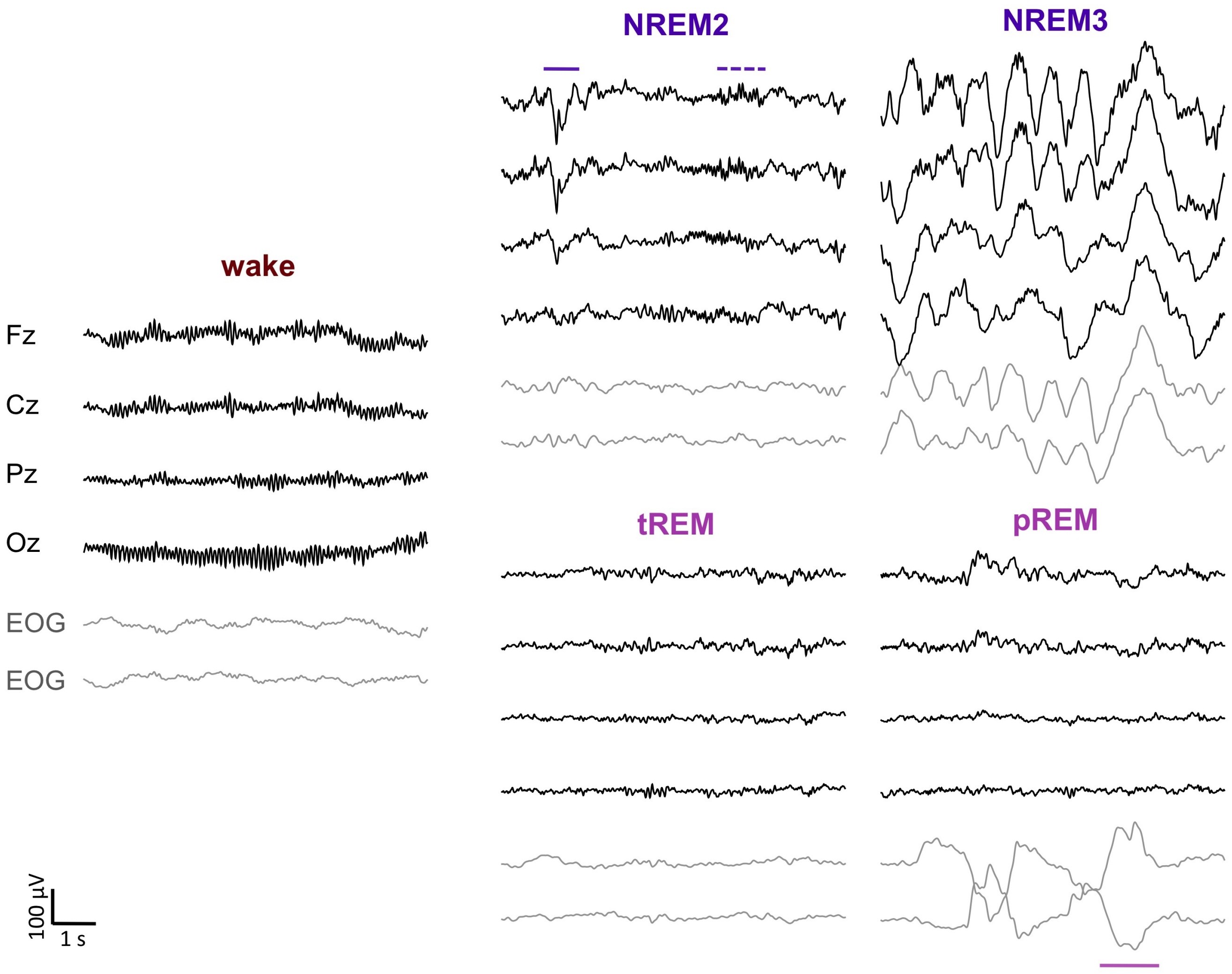

Supplementary Figure 1: EEG and EOG associated to wakefulness, NREM-sleep and REM-sleep

Segments of EEG and EOG data from the same representative individual. Each subpanel shows the EEG activity along the midline (black curves, from top to bottom: Fz, Cz, Pz, Oz) along right and left EOG derivations (gray curves). Wakefulness is here characterized by a strong alpha rhythm (when participants had their eyelids closed) while consolidated NREM sleep (NREM2 and NREM3) is characterized by the presence of NREM sleep hallmarks (example of K-complex: purple line; example of sleep spindle: dashed purple line). Note the apparition of trains of slow-waves in NREM3. REM sleep in turn seems more similar to wakefulness but alpha oscillations are absent despite the fact that sleepers have their eyelids closed. We divided REM-sleep in phasic (pREM) and tonic (tREM) REM sleep based on the presence or absence of REMs (example of REM: fuchsia line). Overall, wakefulness, NREM-sleep and REM-sleep drastically differ in terms of spontaneous brain activity.

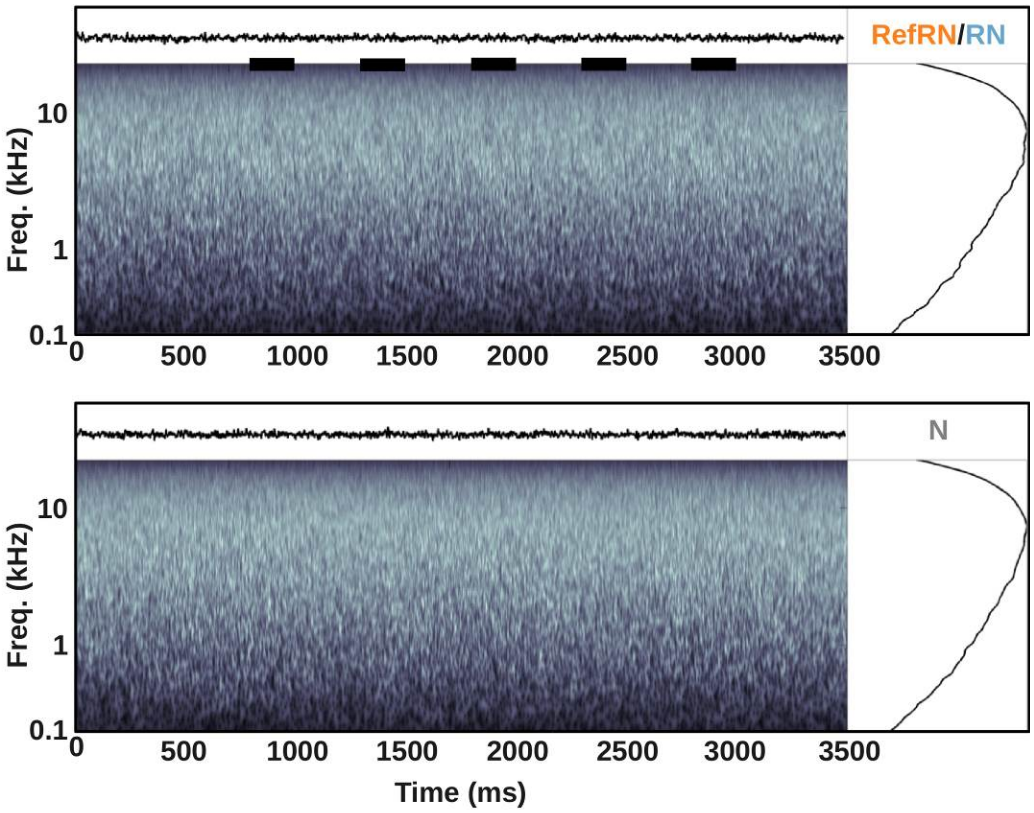

Supplementary Figure 2: Modeled cochlear representation of RefRN/RN and N stimuli
Output of a peripheral auditory model (Spectro-Temporal Excitation Pattern[1]) for noise exemplars either presenting repeated segments (RN/RefRN, top) or not (N, bottom). The model simulates the cochlear filter, providing an estimate of the information transmitted to the central nervous system. In the RefRN/RN condition, the positions of repeated segments are indicated with black horizontal bars. See also the Supplementary Audio 1 and 2 for audio exemplars.

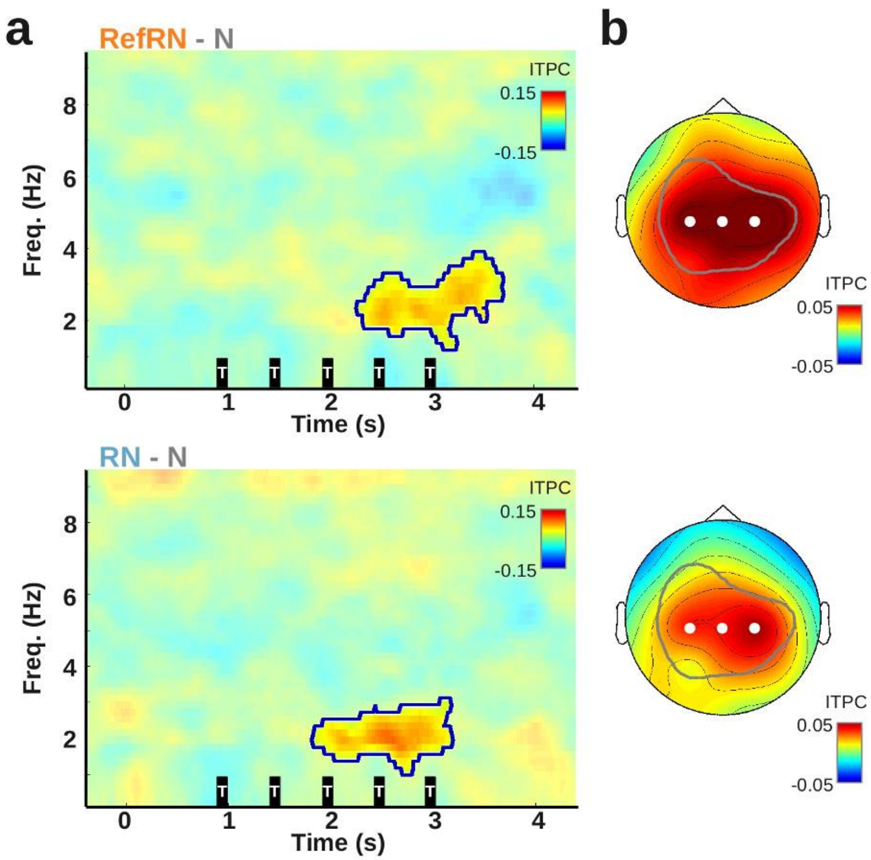

Supplementary Figure 3: Noise-repetitions are associated with an increase in Inter-Trial Phase Coherency (ITPC)
(a) Time-frequency decomposition of the difference in ITPC between RefRN (top) or RN (bottom) and N trials. ITPC was computed over central electrodes (C3, C4, Cz: white circles in panel b) and averaged across participants (N = 20) for the Pre-Sleep phase (participants awake and responsive). Purple contours show clusters surviving a Monte-Carlo permutation test ($p_{cluster} < 0.05$). (b) Scalp topographies of ITPC values for the RefRN (top) and RN (bottom) conditions when averaged over the corresponding clusters in panel a. Gray contours show, for comparison, the distribution of the RefRN vs. N effect observed in evoked-potentials (Memory-Evoked Potentials, MEP, see Figure 2b).

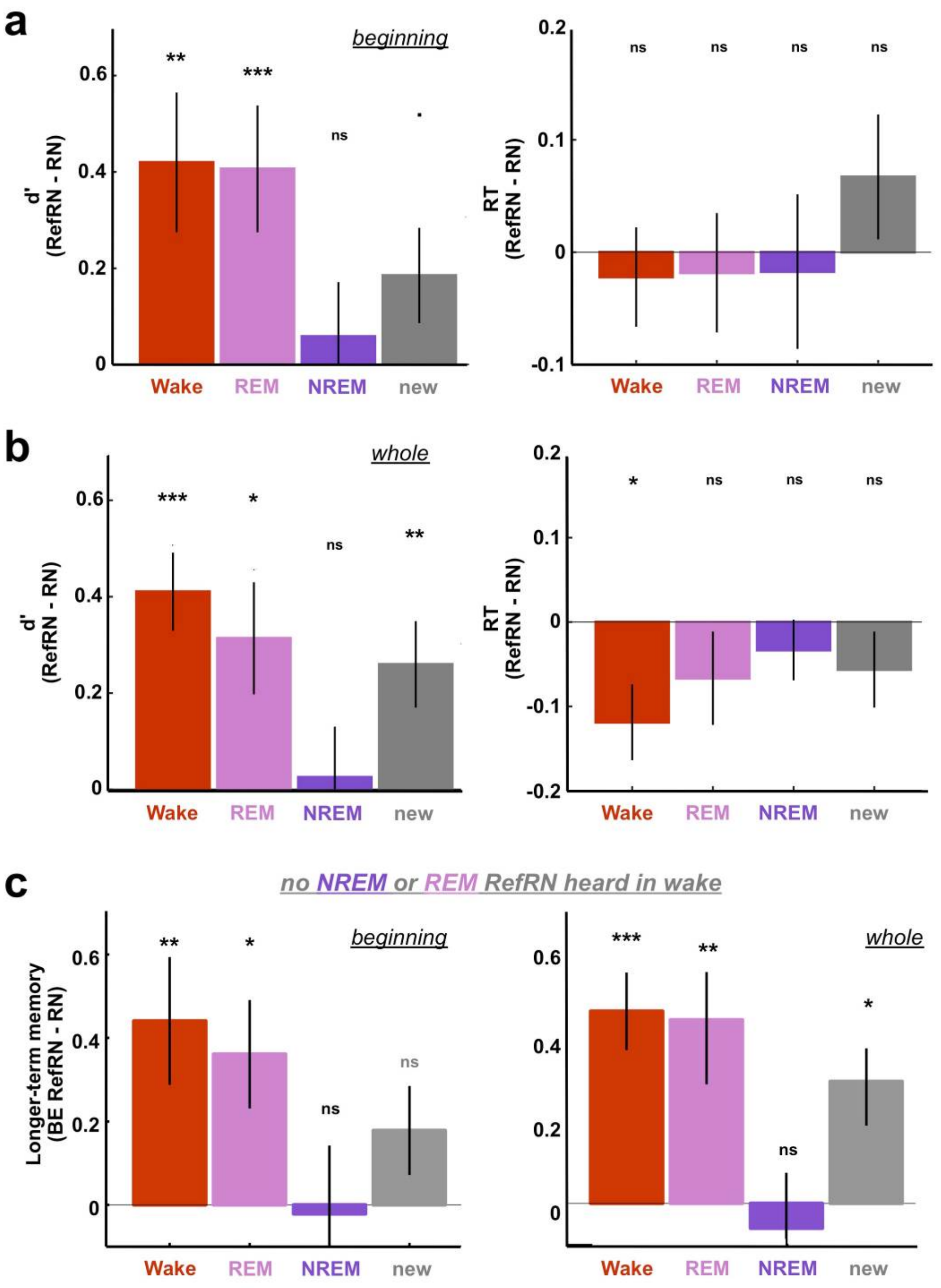

Supplementary Figure 4: The positive effect of REM-sleep and negative effect of NREM-sleep in the Post-Sleep phase

(a) Behavioral efficacy (BE) combines participants' accuracy (d') and rapidity (RT). We show here the RefRN-RN difference for d' and RT separately at the beginning of the Post-Sleep Memory-Test (see Figure 3a left). Here and below, bar-plots show the mean value across participants (N=20) and error-bars the standard-error of the mean. Stars atop bars indicate the results of the statistical tests (t-tests against 0, $p<0.001$: ***; $p<0.01$: **; $p<0.05$: *, ns: $p\geq0.05$). (b) Same as panel a but for the entire Memory-Test (see also Figure 3a right). (c) Figure 3a shows the RefRN vs. RN contrast on the behavioral efficacy (BE) index computed in the Post-Sleep phase. In particular, we showed in Figure 3a an increase in performance for RefRN items heard in REM-sleep. To check that this effect is not due to the few RefRN targets that were presented around (micro)-awakenings, the very same analysis was performed, this time excluding these items (see Methods). BE was computed separately for RefRN heard during wakefulness, REM, NREM or new RefRN. Note that the exact same pattern of results was observed as in Figure 3a ruling out the possibility that our effect is driven by the RefRN targets heard around (micro)-awakenings.

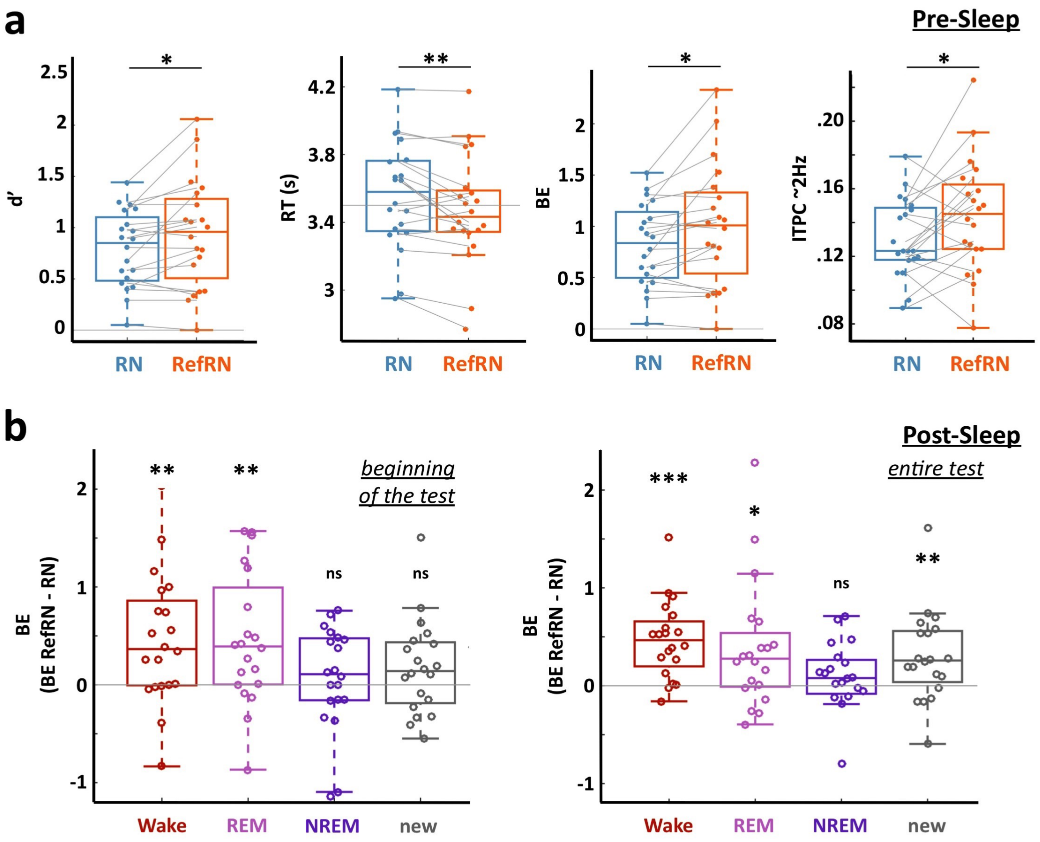

Supplementary Figure 5: Individual data for the behavioral and electrophysiological indexes of learning

Panel a illustrates the same data as the bar graphs in Figure 2 (from right to left: accuracy (d'), reaction times (RT), behavioral efficacy (BE) and inter-trial phase coherency (ITPC) around 2Hz) but shows individual data-points. Box-plots show the median (central line within the boxes), 25th and 75th percentile (lower and upper edge of the boxes) of data-points distribution. Whiskers show the extreme values not considered as outliers (see boxplot.m function in Matlab for details). Individual data-points are plotted along the box-plots (circles). Stars atop graphs refer to the RefRN vs. RN comparison (paired t-test, here and below: $p<0.01$: **; $p<0.05$: *). Lines between circles link the values corresponding to the same participant in both conditions. Note that, despite the rather large inter-subject variability, there are clear paired differences between the RefRN and RN conditions. Panel b illustrates the same data as Figure 3a (Behavioral efficacy indexes of longer-term memory (RefRN - RN) computed for the beginning or the entire Post-Sleep blocks) but shows again individual data-points. Stars atop boxes indicate the results of the statistical tests (t-tests against 0, $p<0.001$: ***; $p<0.01$: **; $p<0.05$: *, ns: $p\geq0.05$).

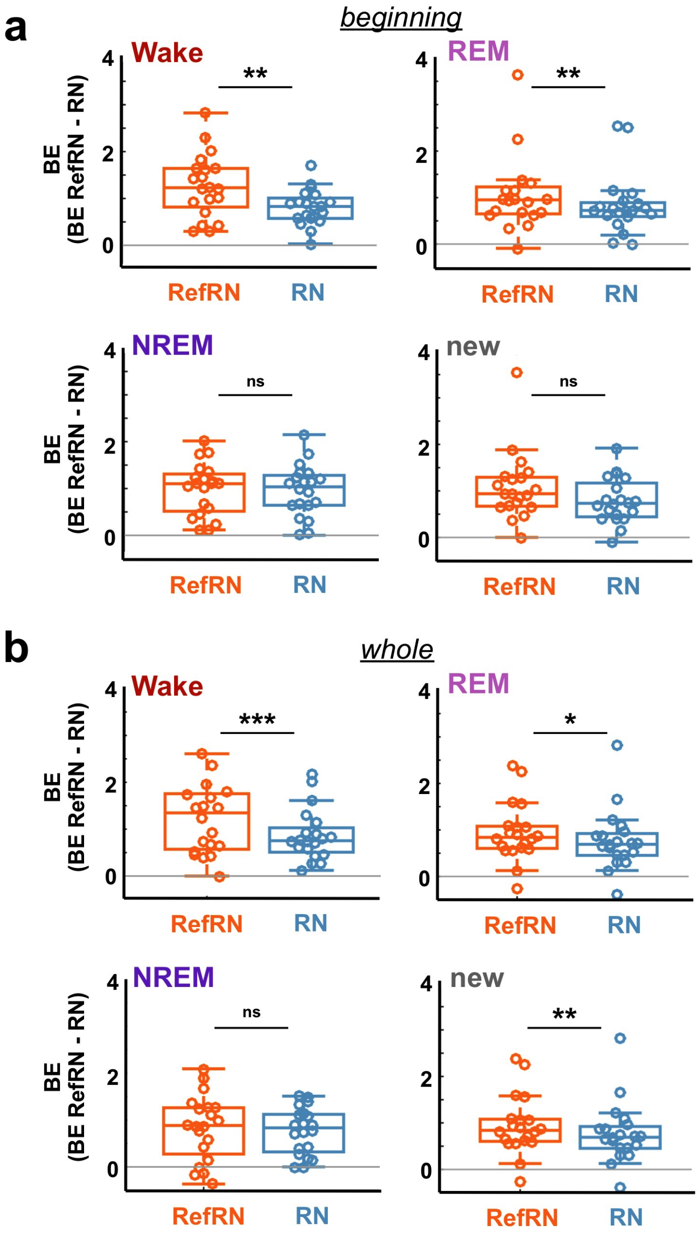

Supplementary Figure 6: Behavioral Efficacy in the Post-Sleep phase for RefRN and RN trials separately
Behavioral Efficacy during the Memory-Test as shown in Figure 3 but for RefRN and RN separately. Panel a shows BE computed at the beginning of the Memory-Test (see Figure 3a, left) and panel b shows BE when considering the entire Memory-Test (Figure 3a, right). Box-plots show the median (central line within the boxes), 25th and 75th percentile (lower and upper edge of the boxes) of data-points distribution. Whiskers show the extreme values not considered as outliers (see boxplot.m function in Matlab for details). Individual data-points are plotted along the box-plots (circles). Stars atop boxes indicate the results of the statistical tests comparing RefRN and RN (paired t-tests, $p<0.001$: ***; $p<0.01$: **; $p<0.05$: *, ns: $p\geq0.05$).

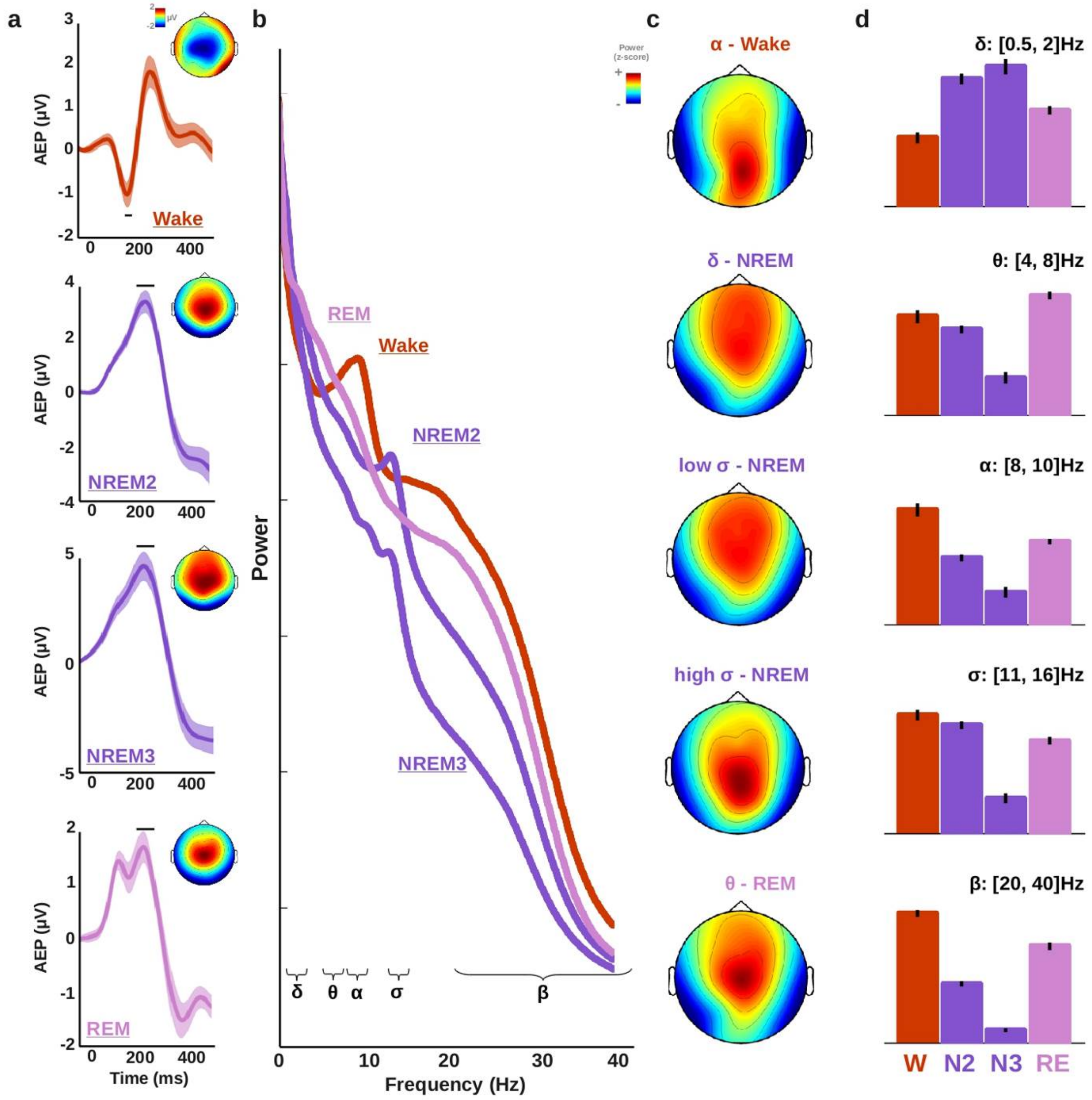

Supplementary Figure 7: Differences in Auditory-Evoked Potentials (AEPs) and spectral profiles across sleep stages (a) Electroencephalographic (EEG) signal time-locked to stimuli onset and averaged across participants (N = 20) for the Sleep phase. Stimuli were collapsed across experimental conditions. Curves show the Event-Related Potentials (ERPs) associated to the silence-to-noise transition. Note the canonical evoked potentials (Auditory-Evoked Potentials, AEPs[2]). These AEPs differed between vigilance states. 1st row: Wake AEP with typical N1 and P2 components; 2nd row: NREM2 AEP with the N1 component replaced by a high-amplitude positive component; 3rd row: NREM3 AEP similar as NREM2; 4th row: REM AEP characterized by the disappearance of the N1 component bringing the preserved P1 and P2 components out. Shaded areas around curves indicate the standard error of the mean (SEM) computed across participants. Insets show the scalp topographies of the component marked with a black horizontal bars. Note that given the time-window considered, these AEPs only reflect the silence-to-noise transition since the window ends before the presentation of repeated targets. (b) Power spectra computed across participants for the Sleep phase using a Fast-Fourier Transform (FFT). Power was normalized and expressed in decibels (dB, see Methods). Note several deviations from the 1/f trend. These increases in certain frequency bands depend on the vigilance state: wakefulness was characterized by an increase within the α band ([8,

10] Hz) and β band ([20, 40] Hz), NREM sleep by an increase within the δ band (< 5 Hz) and σ band ([11, 16] Hz) and REM-sleep by an increase within the θ band ([4, 8] Hz). (c) Scalp distributions of the power in different frequency bands for the appropriate vigilance state (wake: α, β; NREM: δ, σ; REM: θ). Power was z-scored across sensors in order to stress the differences in the spatial distribution of these brain rhythms. (d) Power averaged across the abovementioned frequency bands and for each vigilance state for electrode Cz. As classically observed, the transition from wakefulness to NREM2 is characterized by the disappearance of α and β rhythms replaced by an increase within the δ and σ bands corresponding to the apparition and multiplication of the NREM-sleep hallmarks that are slow-waves and sleep-spindles. Transition to REM-sleep is characterized by the partial recovery of α and β rhythms as well as high θ power. Thus the vigilance states here investigated correspond to drastically different brain states.

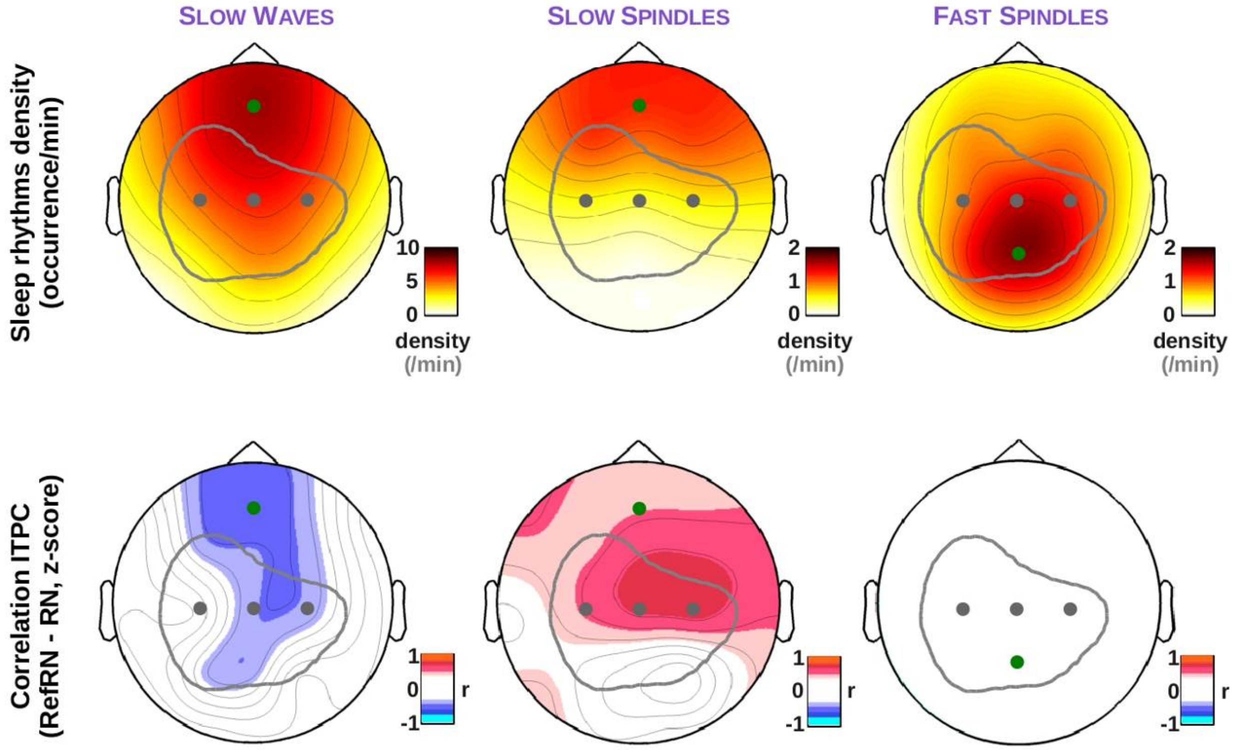

Supplementary Figure 8: Correlations between the amount of slow-waves, fast and slow spindles recorded overnight and the EEG learning index upon awakening
1st row: scalp distribution of slow-waves (left), slow-spindles (spindle frequency: [11, 13] Hz, middle) and fast-spindles (spindle frequency: [13, 16] Hz, right) densities as detected using dedicated algorithms (see Methods for details). Note the typical frontal predominance of slow-waves and slow spindles and the centro-parietal topography of fast spindles. Green dots show the electrodes with the highest densities: Fz for slow-waves and slow spindles, Pz for fast spindles. 2nd row: scalp distribution of Pearson's correlation coefficients computed between the EEG learning index upon awakening ($ITPC_{RefRN}$ - $ITPC_{RN}$) and slow-waves (left), slow-spindles (middle) and fast spindles (right) densities (across participants). Non-significant ($p>0.05$, uncorrected) coefficients are displayed in white. Note that the EEG learning index was positively correlated with the density of slow-waves and negatively correlated with frontal spindles. Gray contours show the distribution of the RefRN vs. N effect observed in evoked-potentials in wakefulness (Figure 2b). Importantly the distribution between learning and spindle density was not centered on the peak of the scalp distribution for the sleep spindles. There was no significant correlation between fast spindles and the EEG learning index (all ps > 0.05).

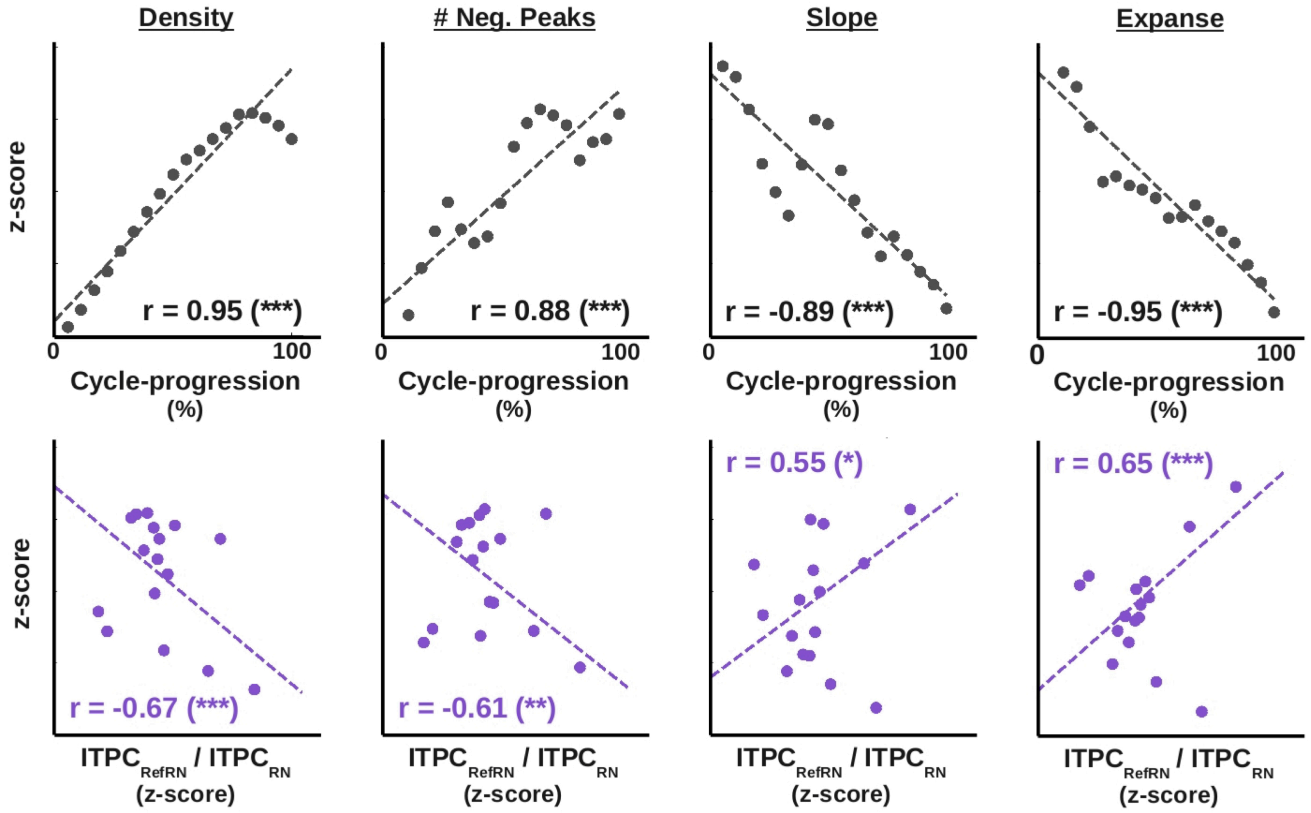

Supplementary Figure 9: Slow-waves characteristics change from light to deep NREM-sleep
1st row: Evolution along sleep cycles of some properties of the slow-waves detected in NREM sleep (NREM2-3, N = 82 cycles in 18 participants, see Methods). Seep cycles were binned in order to normalize their length. Progression within the cycles is expressed in percentage (see also Figure 7). While density and the number of negative peaks increased within cycles, the slope and spatial expanse (i.e. proportion of sensors affected by a given slow-waves) decreased. 2nd row: Correlations between the slow-waves parameters and the EEG learning index ($ITPC_{RefRN}$ / $ITPC_{RN}$) computed as in Figure 7b. The learning index was negatively correlated with slow-waves' density and the number of negative peaks but positively correlated with slow-waves slope or spatial expanse. Thus, the suppressive effect observed in deep NREM-sleep corresponds to the appearance of more numerous but also more local slow-waves (type-II slow-waves[3]). Pearson's correlation coefficients were computed between each slow-waves parameter and either cycle-progression (top) or ITPC (bottom). These coefficients are displayed on each graph. Dotted lines show linear regressions between the pairs of variable. Stars indicate the significance level of the correlation coefficients (*: $p < 0.05$, **: $p < 0.01$, ***: $p < 0.001$).

	Wake	NREM			REM		total
		NREM1	*NREM2*	*NREM3*	*tREM*	*pREM*	
duration (min.)	220 (9.4)	50.1 (4.7)	200 (8.4)	110 (6.8)	49.2 (3.3)	29.8 (3.4)	659 (8.7)
%	33 (1.31)	7.5 (0.73)	30 (1.18)	16 (0.90)	7.4 (0.52)	4.4 (0.49)	

Supplementary Table 1: Summary of the sleep scoring

Sleep was scored as wakefulness, NREM (Non-Rapid-Eye Movement) sleep stage 1 (NREM1), NREM-sleep stage 2 (NREM2), NREM-sleep stage 3 (NREM3), tonic REM-sleep (tREM) and phasic REM-sleep (pREM) according to established guidelines[4]. Time spent in each sleep stage is shown in minutes (1st row) and percentage of the recordings' duration (2nd row). Numbers refer here to the average across participants while numbers in brackets refer to the standard error of the mean computed across participants (N = 20). The Pre-Sleep and Post-Sleep phases were here included (entire recording session), which explain the extent of the wake periods.
